# High-Resolution Modeling of Transmembrane Helical Protein Structures from Distant Homologues

**DOI:** 10.1371/journal.pcbi.1003636

**Published:** 2014-05-22

**Authors:** Kuang-Yui M. Chen, Jiaming Sun, Jason S. Salvo, David Baker, Patrick Barth

**Affiliations:** 1 Verna and Marrs McLean Department of Biochemistry and Molecular Biology, Baylor College of Medicine, Houston, Texas, United States of America; 2 Department of Pharmacology, Baylor College of Medicine, Houston, Texas, United States of America; 3 Structural and Computational Biology and Molecular Biophysics Graduate Program, Baylor College of Medicine, Houston, Texas, United States of America; 4 Howard Hughes Medical Institute and Department of Biochemistry, University of Washington, Seattle, Washington, United States of America; Icahn School of Medicine at Mount Sinai, United States of America

## Abstract

Eukaryotic transmembrane helical (TMH) proteins perform a wide diversity of critical cellular functions, but remain structurally largely uncharacterized and their high-resolution structure prediction is currently hindered by the lack of close structural homologues. To address this problem, we present a novel and generic method for accurately modeling large TMH protein structures from distant homologues exhibiting distinct loop and TMH conformations. Models of the adenosine A2AR and chemokine CXCR4 receptors were first ranked in GPCR-DOCK blind prediction contests in the receptor structure accuracy category. In a benchmark of 50 TMH protein homolog pairs of diverse topology (from 5 to 12 TMHs), size (from 183 to 420 residues) and sequence identity (from 15% to 70%), the method improves most starting templates, and achieves near-atomic accuracy prediction of membrane-embedded regions. Unlike starting templates, the models are of suitable quality for computer-based protein engineering: redesigned models and redesigned X-ray structures exhibit very similar native interactions. The method should prove useful for the atom-level modeling and design of a large fraction of structurally uncharacterized TMH proteins from a wide range of structural homologues.

## Author Summary

Membrane proteins perform crucial cellular functions and can be involved in serious diseases but remain difficult to study experimentally. Hence, high-resolution membrane protein structures are scarce which hinders the design of selective therapeutics and of receptors with novel function for systems/synthetic biology applications. The computational modeling of membrane protein structures represents an important alternative approach but, to achieve high accuracy, usually requires structural information from closely related proteins currently unavailable for most membrane proteins. To address this limitation, we have developed a novel method to predict membrane protein structures from the structures of non-closely related proteins that differ both in loop and transmembrane regions. Using this approach, we show that a large diversity of membrane proteins can be reconstructed at a level of accuracy suitable for computer-based protein engineering applications. Because requiring information from a single distant homolog only, we expect that around 60% of human membrane proteins can reliably be modeled using our approach, thereby allowing precise structure/function studies on a large fraction of structurally uncharacterized membrane proteins.

This is a PLOS *Computational Biology* Methods article.

## Introduction

Membrane proteins perform a wide diversity of critical functions in living cells but are also involved in serious diseases and represent more than 60% of current drug targets [1], [2]. Despite recent tremendous progress in membrane protein expression, biochemistry and X-ray crystallography, eukaryotic membrane protein structures remain difficult to characterize experimentally [3]. The lack of high-resolution structures hinders the design of more effective therapeutics and of receptors with novel function for systems/synthetic biology applications which rely on atomic-resolution information [4]. The high-resolution prediction of membrane protein structures is therefore an important alternative approach but remains a major challenge in absence of close structural homologues [5]. Although numerous methods have been developed to model G protein-coupled receptor (GPCR) structures [6]–[9], much fewer techniques have been developed and applied to the entire class of alpha helical membrane proteins. Current state-of-the-art *de novo* structure prediction techniques of alpha helical membrane proteins can generate low-resolution models with native-like topologies [10]–[12] and, despite some insightful applications [13], most current comparative modeling methods do not significantly improve starting templates [14], [15]. The main structural differences between distant homolog transmembrane alpha-helical (TMH) proteins are found in loop regions and in helical conformations shaping TMH core structures and ligand/effector binding sites. While the problem of rebuilding protein loops has been extensively studied [16], [17], the accurate modeling of membrane protein structures from distant homologues diverging in both loop and TMH core regions is a remaining unsolved challenge [5]. The origins of TMH conformational diversity are multiple and range from the presence of localized sequence-specific distortions (e.g. Proline-induced kinks) to local bends and global tilts stabilized by specific tertiary contacts [18]–[23]. Many of these features cannot be accurately predicted from sequence information alone and requires the explicit modeling of atom-level physical interactions stabilizing these structures [18]–[20], [24]. The large size of TM proteins and associated number of degrees of freedom combined with the ruggedness of the all-atom energy landscape make their prediction at atomic resolution computationally intractable using an exhaustive conformational search in torsional angle space.

To address this problem, we have developed a general modeling strategy based on efficient sampling techniques of alternative TMH structures to reconstruct both TMH core and loop regions from distant structural homologues. The method was stringently validated in two blind predictions where the generated models were top-ranked [14], [15] and in a large benchmark dominated by pairs of membrane protein distant homologues where starting templates were almost all significantly improved. Computational design calculations suggest that the models should be of suitable accuracy for rational protein engineering applications.

## Results

### Approach

**Figure 1:**
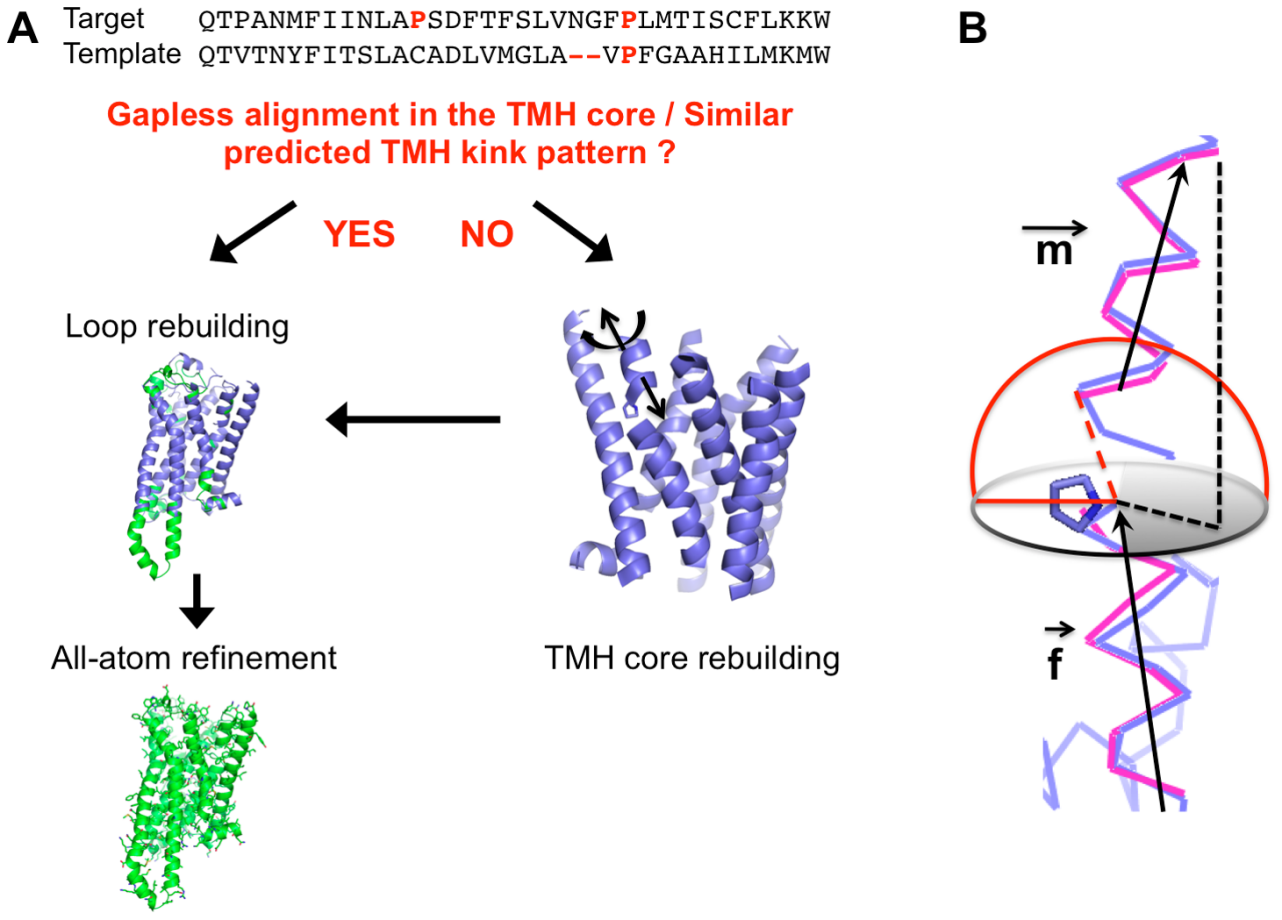
General framework for the high-resolution modeling of membrane protein structures from structural homologues. **A.** Sequence alignment between target and template sequences in a TMH region revealing gaps and potentially different proline-induced distortion patterns in which case TMH core structure is rebuilt by sampling alternative rigid-body conformations of all or selected TMHs (Methods) before loops are rebuilt de novo and the fully-reconstructed structure is refined at all-atom (right). If no gaps and identical distortion patterns are identified between template and target, no TMH rebuilding is performed and loop rebuilding+refinement protocol is performed (left). **B.** Schematic representation of the sampling of kinked TMH conformations. A kinked helix is defined by 3 regions: An N- and C-terminal helix fragment separated by a distorted bend region (typically 4 to 6 residues N-terminal to a Proline for example) which can adopt a large diversity of local structures (3_10_ to π turns) and is modeled de novo using fragment insertion techniques. Each helix fragment is treated as a rigid-body and defined by a helical axis (m for “moveable” and f for “fixed” defining the reference state). The m helix is moved with regard to the f helix according to the following degrees of freedom: 1) Distance between the C-terminal position of the m helix and the N-terminal position of the f helix (dotted red line allowing to sample a hemispherical surface shown in red). 2) Tilt angle between the m and f vectors sampled so that the projection of the N-terminal position of the m helix (black dotted line) on a plane orthogonal to the f axis and crossing the proline ring preferentially occupies the semicircle (grey) away from the proline ring (see methods).

As shown in **Fig. 1**, multiple sequence alignments using Hidden Markov Model (HMM)-based techniques [25] are first performed to identify structural homologues that best align with the target sequence. The quality of the alignment in the TMH regions leads to two different model rebuilding strategies: 1) If the alignment in the TMH regions does not exhibit significant gaps, if the positions of coils or residues promoting local distortions are identical and if TMHs are predicted to have similar length, then target and template TMH structures are likely very similar (**Methods**). In this situation, the template TMH structure is first kept fixed onto which loops diverging between target and template are reconstructed *de novo* using fragment insertion techniques [10], [16]. The reconstructed models are then refined at the all-atom level [24] (**Fig. 1**). 2) If one of the above-mentioned conditions is not satisfied however, target and template TMH structures may differ significantly and the target TMH region is also reconstructed as described below (**Fig. 1**). TMH structures mostly sit in the hydrophobic environment of the lipid membrane disfavoring any unsatisfied polar atom. Therefore, we reasoned that, except in local bends or kinked regions where hydrogen-bond networks may be partially disrupted, most TMH regions to be rebuilt adopt helical conformations. Previous work also suggests that most bent helices can be approximated by straight TMH fragments away from the local distortion [24] which can adopt diverse structures (from a 3_10_ turn to a π helix) [18], [19], [23]. To efficiently identify alternative low-energy TMH conformations, each TMH fragment away from local predicted bends (that usually span from 4 to 6 residues) is first modeled as a rigid-body helix and its conformation optimized in a low-resolution search sampling helical rigid-body degrees of freedom (see Method). This low-resolution search averages out side-chain conformations, effectively flattening the conformational free energy landscape and allows the rapid identification of low-energy TMH conformations [10] with alternative interhelical and/or kink angles. Loop and local TMH regions around bends or kinks are then rebuilt using fragment insertion techniques and the fully-reconstructed low-resolution structures are refined at all-atom to identify the lowest-energy native-like structures. At this stage, global deformations of TMH stabilized by short-range atom-level tertiary interactions can be identified and selected by energy [24]. To avoid sampling regions of the conformational space unlikely to be occupied by the peptide chain, distance constraints are applied to the template structure at pairs of residues in proximity and conserved in both target and template sequences (**Methods**).

### Only distant structural homologs are available for a large fraction of human transmembrane helical proteins

To assess the significance of our technique developed to model membrane proteins from distant homologs, we analyzed the space of structural homologs available to all human TMH proteins using HHpred [25]–[27], a toolkit for searching and aligning query sequences with sequences from existing structures. The resultant HHpred alignments were filtered by a range of percent sequence identity thresholds (i.e. of homolog hit versus target) and percent coverages (i.e. of total length of target sequence) of 90%, 75%, 60% or 50%. As shown in **Fig. S1A**, the percentage of human multi-pass TMH proteins sharing 15–25%, 25–35% and >35% sequence identity with their best structural homolog hit is 43%, 12% and 12%, respectively. Similar distributions were obtained for datasets including also human single-pass TMH proteins or consisting of human multi-pass TMH proteins truncated to their TM domains (**Fig. S1C,D**). These results indicate that only distant structural homologs are currently available for a large fraction of human TMH proteins. Moreover, as shown in **Fig. S1B**, only one single distant structural homolog is found for a large fraction of these TMH proteins. These results justify our approach and led us to test our technique on a benchmark where membrane protein structures were primarily modeled from single distant structural homologs.

### Submitted models of the adenosine receptor (A2AR) and chemokine receptor (CXCR4) were first-ranked in blind prediction GPCR-DOCK contests in the receptor structure accuracy category

**Figure 2:**
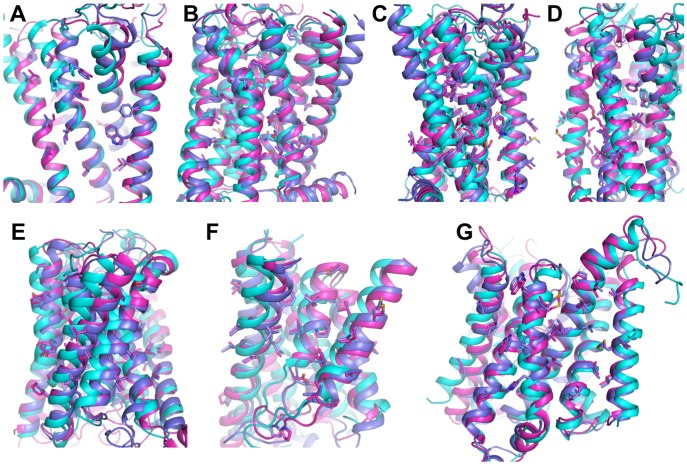
Accurate prediction of TMH structures. Superposition of selected models, templates and native structures are in magenta, cyan and blue, respectively with backbone in cartoon and side-chains in stick. **A.** Blind prediction of adenosine A2A receptor (3EML) from the beta1 adrenergic receptor (2VT4). **B, C.** Modeling of the beta2 adrenergic receptor (2RH1) from the chemokine CXCR4 receptor (3ODU). **D.** Modeling of the beta2 adrenergic receptor (2RH1) from bovine rhodopsin (1U19). **E.** Modeling of the Ammonia Channel AmtB (1U7G) from Rhesus protein Rh50 (3B9W). **F.** Modeling of ECF-type riboflavin transporter (3P5N) from ECF-type ABC transporter thiamine-specific S-component ThiT (3RLB). **G.** Modeling of BtuCD protein (1L7V) from (ATP)-binding cassette ABC transporter (2NQ2).

**Figure 3:**
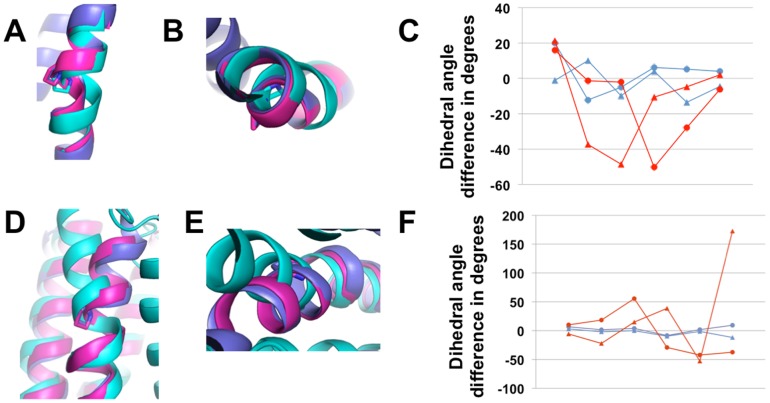
Accurate prediction of distorted helical structures. Superposition of selected models, templates and native structures are in magenta, cyan and blue, respectively **A–C.** Blind prediction of CXCR4 from B2AR. **A, B.** Cartoon representations of TMH2. **C.** Deviation in backbone dihedral angles (phi, triangles; psi, circles) between model (blue) or template (red) and native structure over the local bend of TMH2 from Pro 92 to Phe 87. **D–F.** Modeling of the adenosine A2A receptor from bovine rhodopsin. **D, E.** Cartoon representations of TMH2. **F.** Deviation in backbone dihedral angles (phi, triangles; psi, circles) between model (blue) or template (red) and native structure over the local bend of TMH2 from Pro 58 to Gly 53.

**Figure 4:**
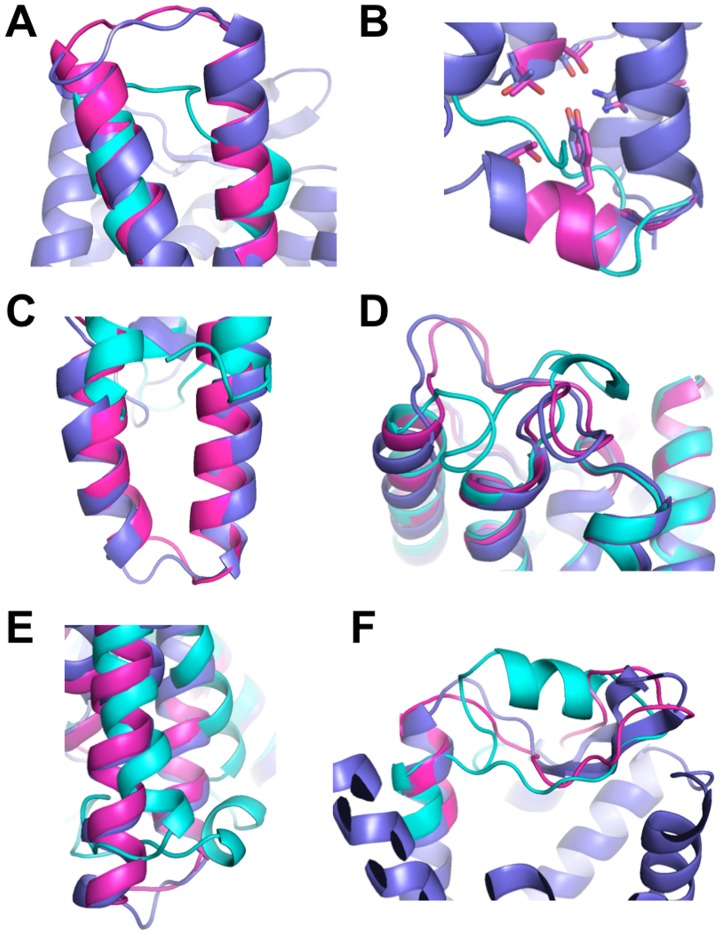
*De novo* prediction of loop structures in membrane proteins. Cartoon representation of selected models, templates and native structures are in magenta, cyan and blue, respectively. **A.** Blind prediction of the chemokine CXCR4 receptor (3ODU) from the beta2 adrenergic receptor (2RH1): extracellular loop 3 (residues G220-I246). **B.** Blind prediction of the dopamine D3DR receptor (3PBL) from the beta2 adrenergic receptor (2RH1): intracellular loop 2 (residues V109-T118). **C.** Modeling of the Squid Rhodopsin (2Z73) from bovine rhodopsin (1U19): intracellular loop 3 (residues N229-N254). **D.** Loop modeling of ECF-type riboflavin transporter (3P5N) from the BioY transporter (4DVE) (residues I55-G83). **E.** Loop modeling of the Ammonia Channel AmtB (1U7G) from the Ammonium Transporter Amt-1 (2B2H) (residues I325-M342). **F.** Blind prediction of the chemokine CXCR4 receptor (3ODU) from the beta2 adrenergic receptor (2RH1): extracellular loop 2 (residues A174-N192).

The technique was tested in two challenging blind predictions of membrane receptor structures, i.e. GPCR-DOCK 2008 for the adenosine receptor (A2AR) [15] and GPCR-DOCK 2010 for the chemokine receptor (CXCR4) [14]. The closest homolog template to A2AR was the beta 1 adrenergic (B1AR) receptor structure [28] sharing 32% sequence identity and exhibiting excellent sequence alignment in the TM region with A2AR. Therefore, TMH remodeling of the template structure was not required. A total of 206 models were submitted by the participants but very few showed significant improvements compared to the initial template structure. Among the top 10 models for both receptor and ligand binding prediction accuracy, one of our submitted models ranked co-first and first for the receptor prediction accuracy over the full length (i.e. 283 residues) and TMH region (i.e. 214 residues), respectively (the reported model from Costanzi had a lower “full-length” RMSD but did not include the entire long ECL2 loop, see Table 1 in [15]). An additional model submitted without ligand (submission 3600_8, Supplementary information in [15]) was even closer to the target (Cα RMSD of 2.9 Å over 283 residues) and ranked first among all submitted models for both TMH and full-length structures with a Z-score of 1.51. For CXCR4, although HHpred identified the beta 2 adrenergic receptor (B2AR) as the best aligned structural homolog, B2AR is a distant homolog sharing only 22% sequence identity with CXCR4 [29] and its second TMH did not align well with the target near a proline-inducing kink. The C-terminal part of TMH2 starting from the kink and the loop structures were therefore remodeled, and 5 low-energy models with docked ligand were submitted. One model was ranked first for the accuracy of the receptor structure among all the 158 models submitted by the participants for the two CXCR4 structures (Z-score = 1.72) and 2 additional ones were ranked second and third for the prediction accuracy of the CXCR4/IT1t structure (Z-scores of 1.36 and 1.24) [14]. Both blind predictions demonstrate that our technique significantly improved starting templates and generated models exhibiting several structural features closer to the target X-ray structures than to the starting template. For example, the TMH shifts in the A2AR structure from B1AR (**Fig. 2A**), the local kink in TMH2 of CXCR4 (**Fig. 3A–C**) and the 27 residues long extracellular loop 3 of CXCR4 (residues G220-I246) (**Fig. 4A**) were predicted quite accurately. Although the conformation of the 16 residues long partially disordered extracellular loop 2 (residues A174-E179, R183- N192) was not predicted with near-atomic accuracy, its conformation was closer to the target CXCR4 than to the starting template (**Fig. 4F**) and was the most accurate prediction for that region among all submitted models [14]. We also attempted the modeling of D3DR but, since close homologs (sequence identity >30%) were available, the main interest for this target was not receptor modeling but ligand docking which is outside the scope of the present study. With a Z-score of 0.41, our best model of D3DR ranked within the top 35% of the population of models. However, the accuracy of the models may not reflect the ability of our method to model the receptor because the ICL3 loop was mistakenly not rebuilt (i.e. the polypeptide chain was not connected between TMH5 and TMH6 due to the presence of T4 lysozyme in the B2AR template), preventing an optimal all-atom refinement of the receptor structure.

### Starting templates are significantly improved in a benchmark dominated by distant homolog membrane protein structure pairs

**Figure 5:**
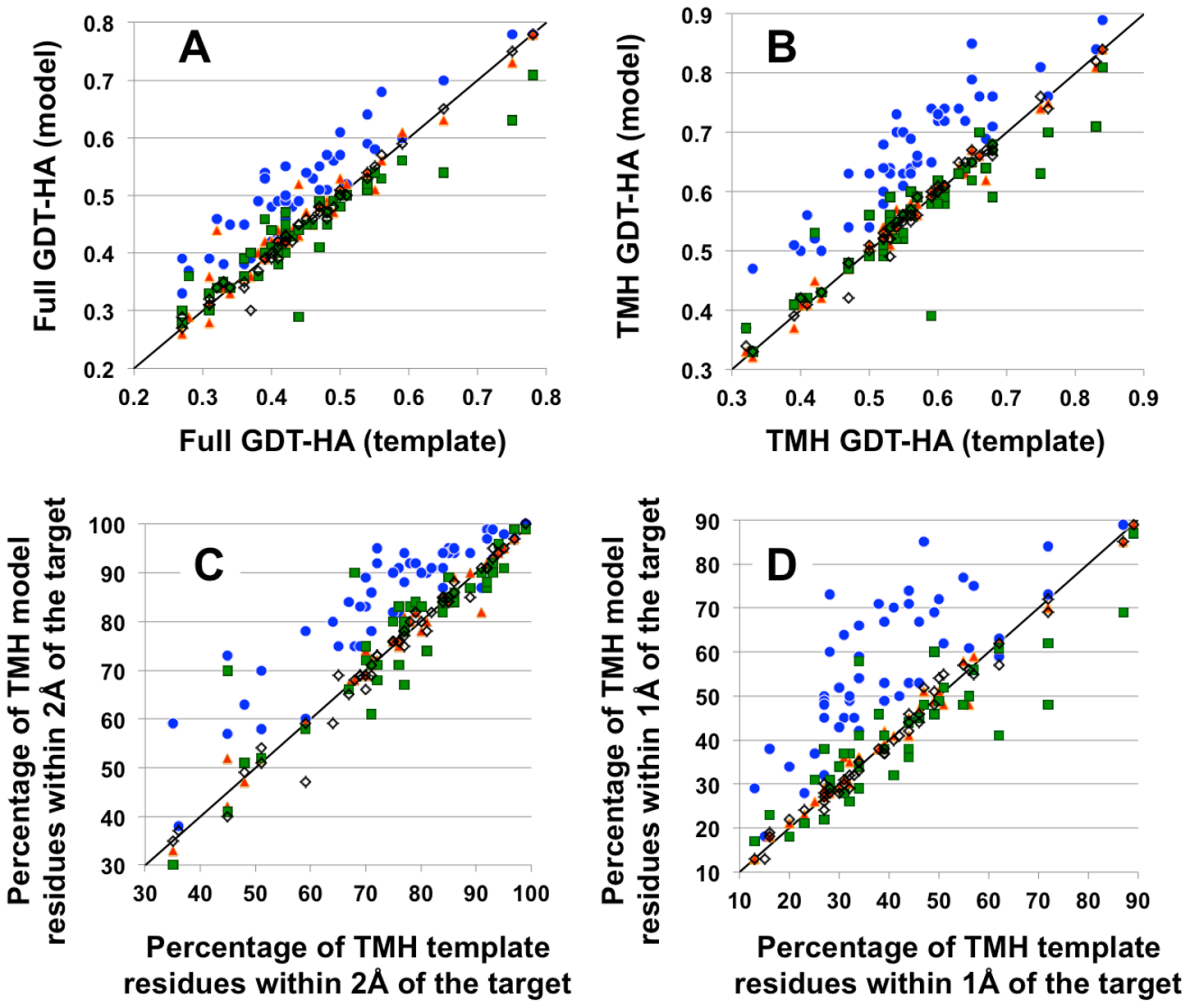
Significant improvements of close to distant homolog templates. In the panels, each dot represents a prediction of a target from a template structure (blue for RosettaMembrane, red for MEDELLER [30], green for I-TASSER [32], [33], black open squares for MODELLER [31]). The accuracy of the model (y-axis) and starting template (x-axis) to the X-ray structure of the target is given for 50 protein pairs. The black line represents the absence of improvements where both model and template have identical accuracy. Accuracies are measured using GDT-HA [34] over full-length (**A**) and TMH (**B**) structures. They are also reported for the TMH regions as the percentage of residues within 2 Å (**C**) and 1 Å (**D**) of the native structure.

To further test whether our method consistently improves homolog templates, we selected a representative dataset of 50 membrane protein structure pairs exhibiting a wide diversity of sequence identity (from 15% to 70%), length (from 183 to 420 residues and topology (from 5 to 12 TMHs) (**Methods**, **Table S1**). In this dataset, 28 pairs were GPCRs (class A or B), 22 pairs were non-GPCRs and 37 pairs were distant homologs sharing not more than 25% of their sequences. In each pair, one structure was assigned as the target to be modeled and the other one as the starting template. 36 pairs exhibited poor sequence alignment for at least one TMH and required both TMH and loop rebuilding prior to all-atom refinement. Specifically, 21 GPCR pairs required sampling alternative conformation of one distorted TMH (**Table S2**), 15 non-GPCR pairs required at least one TMH to be rebuilt and the Lac permease/EmrD pair sharing only 15% sequence identity required all TMHs to be simultaneously rebuilt (**Table S1**). The models were selected by all-atom energy and clustering (**Methods**). The quality of the predictions was analyzed for their accuracy over the full-length, TMH structures and individual distorted TMH conformations. They were compared to the starting template and to models generated with the same input information (e.g. alignment, template structure) using **1.** MEDELLER [30], a comparative modeling technique developed for membrane proteins, **2.** the widely used MODELLER comparative modeling method [31] and **3.** I-TASSER, a widely-used protein structure prediction server [32], [33]. As shown in **Fig. 5** and **Table S1**, our method significantly improves starting templates for all but 4 protein pairs over the full length structure and for all but 3 protein pairs over the TMH regions. The average improvements as measured by GDT-HA over the entire dataset (i.e. High Accuracy Geometric Distance Test measuring similarity between two protein structures [34]) are 0.07±0.04 and 0.10±0.05 for the full length structure and the TMH regions, respectively, and are statistically significant (p values <0.005 and <0.0001, respectively, as measured by student t-test). These improvements are particularly noticeable in the TMH regions where the percentage of residues lying within 1 Å of the native structure is increased by 17±10% thereby decreasing the Cα RMSD from 2.1±0.7 to 1.7±0.7 Å in these regions. In contrast, the models generated by MEDELLER, MODELLER and I-TASSER remain very close to the starting templates and do not exhibit significant improvements as measured by GDT-HA over TMH regions: 0.002±0.01, −0.006±0.05 and −0.01±0.05, respectively (p values >0.5; **Fig. 5**, **Table S1**).

The absence of improvements in the TMH regions was observed for 3 close homolog pairs: 3PBL from 3EML, 4EJ4 from 3RZE and 2IC8 from 2NR9. For 3PBL from 3EML, the template is already very close to the target structure (Cα RMSD = 1.1 Å). At this level of structural similarity, inaccuracies in the energy function and the lack of explicit modeling of buried water molecules, lipids and ligands in the current method may impede further significant improvements.

### Distorted TMHs are modeled with atomic accuracy

**Table S2** summarizes the local improvements on the distorted TMH2, which was rebuilt in 21 GPCR pairs because of the poor sequence alignment between the target and the template in that region. The overall conformation of the kinked TMH2 was improved for all but two pairs as measured by GDT-HA which increased from 0.79±0.05 to 0.86±0.06 and by Cα RMSD which decreased from 1.33±0.42 to 0.84±0.33 Å. Importantly, as shown for 3ODU from 2RH1 (**Fig. 3A–C**) and for 3EML from 1U19 (**Fig. 3D–F**) and in **Table S2**, the precise conformation of the kinked regions that were rebuilt *de novo* was also improved as measured by the differences in dihedral angles between template or model and native structures. When averaged over the bend (i.e. 5 residues, **Methods**), these differences decreased from 26±13° and 34±19° to 15±7° and 14±10°, for phi and psi backbone dihedral angles, respectively.

Modeling the unusually distorted TMH2 of squid rhodopsin (2Z73) [35] was challenging. Proline 90 perturbs and partially breaks the hydrogen bond network between the backbone nitrogen and carbonyl groups of residues 85 to 90 which form a wide π turn splitting TMH2 in two helical fragments. In addition to the wide π turn, the relative position of these helical fragments is also unusual. Unlike many kinked helices [21], the interhelical (kink) angle is only 21 degrees and the C-terminal helix is displaced outside of the TMH core compared to the N-terminal helix, a conformation stabilized by the beta-strand forming an extracellular “lid” over the retinal binding site. In absence of this loop region during TMH rebuilding, the native conformation of the C-terminal helix is not stabilized by a large number of physical contacts with the rest of the TMH core making the selection of that conformation difficult by energy alone. Although our protocol improved starting templates overall, we expect that rebuilding TMH core and loop regions simultaneously may become a more effective strategy for helical conformation stabilized by loop regions and will be explored in future work.

### The largest improvements are mainly observed for the most distant homolog templates

The largest improvements in full length structure and TMH regions (defined as GDT-HA increases ≥0.12) were mainly observed for distant homologues and include both GPCRs and non-GPCRs: 1U19 from 3ODU, 2RH1 from 1U19, 2CFQ from 2GFP and 3P5N from 3RLB. GDT-HA increases ≥0.12 in the TMH region were also mainly observed for distant homologs, such as 1U19 from 2Z73 or 3EML, 2RH1 from 2Z73 or 3ODU, 2Z73 from 3ODU, 3PBL from 3ODU, 3V2Y from 3RZE, 3EML from 3UON, 1U7G from 3B9W, 3P5N from 4DVE, 3V5U from 4KPP and 3GD8 from 3KLY.

Within the GPCR targets, modeling the beta2 adrenergic receptor (2RH1) from bovine rhodopsin (1U19) led to the largest improvements in GDT-HA: 0.13 and 0.19 over the full-length and TM structures, respectively. Although these 2 GPCRs share only 20% sequence identity in the modeled regions, 73% of the model residues lie within 1 Å of the native TM structures compared to only 28% for the starting template and display very similar side-chain conformations compared to in the native structure (**Table S1**, **Fig. 2B,C**). Most of the residues not predicted at atomic resolution belong to the extracellular part of the first TMH which, unlike in 1U19, is poorly packed to the rest of the TM structure in the B2AR crystal structure and is difficult to predict accurately.

Within the non-GPCR targets, the largest improvements in GDT-HA were observed for the ECF-type riboflavin transporter (3P5N) from thiamine-specific S-component ThiT from an ECF-type ABC transporter (3RLB). Although these 2 transporters share only 15% sequence identity in the modeled regions, the overall fold is conserved. Three TMHs poorly aligned with the template were rebuilt leading to 0.14 and 0.16 improvements in GDT-HA over the full-length and TM structures, respectively. In contrast to the template, most of the TMH region in the selected Rosetta model is superimposable to that of the target allowing a large fraction of side-chains to adopt similar packing than in the native structure (**Fig. 2F**). Similar improvements of starting templates leading to close to atomic accuracy backbone and near-native side-chain conformation predictions in the TM region were observed for other distant homolog pairs such as 2RH1 from CXCR4 (Cα RMSD of 1.7 Å, **Fig. 2D**) and 1U7G from 3B9W (Cα RMSD of 1.1 Å, **Fig. 2E**).

Although most of the largest improvements were obtained for distant homologs, the method was also able to improve starting templates for most of the closer homolog pairs that are structurally more similar. For example, improvements in GDT-HA ≥0.1 were observed for the pairs 1U19 from 2Z73, 2Z73 from 1U19, 1J4N from 1FX8, and 1L7V from 2NQ2 sharing more than 25% sequence identity in the modeled regions (**Table S1**). In the latter, reconstruction of distorted TMHs with different kink patterns between target and template allowed accurate prediction of backbone and side-chain conformations in the TM region (Cα RMSD of 1.1 Å, **Fig. 2G**).

### *De novo* prediction of loop structures

When loop sequences are well aligned between template and target, their structures from the template are, as for TM regions, accurately refined at all-atom. In absence of significant sequence alignment with the template, loops are rebuilt *de novo* from sequence and accurately predicting their structures remains a challenge in the field of protein modeling. Three scenarios are typically encountered: 1) When loops are short (typically <8 residues) (e.g. kinks in distorted TMHs) or 2) When loops are long (typically ≥8 residues) but not only composed of disordered segments (i.e. incorporating a significant fraction of secondary structure elements), our approach can rebuild these regions from sequence with near-atomic accuracy (Cα RMSD within 2.5 Å). Examples include the blind-predicted extracellular loop 3 of CXCR4 (residues G220-I246) and the blind-predicted intracellular loop 2 of DRD3 (residues V109-T118) as well as several GPCR and non-GPCR loops in our benchmark (**Fig. 4A–E**). 3) Loops such as the extracellular loop 2 of GPCRs can be long and mostly disordered and/or make numerous contacts with small molecules or with other subunits in the crystal structures. Because crystal contacts or ligands are not modeled by the current method, near-native conformations of loops stabilized by such contacts are very difficult to select by energy alone. Therefore, although our blind predicted model of the long disordered extracellular loop 2 of CXCR4 was significantly more accurate than any other submitted model in the blind prediction, future developments (e.g. integrated loop modeling and ligand docking) will be necessary to consistently reach high-accuracy prediction in these regions and allow accurate prediction of ligand-bound conformations. Nevertheless, our results suggest that our method should be useful in rebuilding and refining X-ray structures of membrane receptors where functionally important loop regions have missing densities or are often deleted to facilitate crystallization.

### Selected models are of suitable accuracy for rational design applications

An important question in the field of protein modeling is the relationship between the accuracy of the models and their potential applications. Near-atomic resolution models should be accurate enough to guide the rational design of mutations and the interpretation of their effects [4]. As a stringent test of the accuracy of our predictions, we subjected the selected models from our benchmark to complete sequence redesign in the TMH regions and compared the results to similar calculations performed with the native X-ray and initial template structures (**Methods**). Single-state design calculations select combinations of amino acids that minimize the free energy of (i.e. predicted to stabilize) the protein. Previous sequence calculations performed on high-resolution transmembrane helical protein X-ray structures recapitulated a significant fraction of native sequences [24], suggesting that this fraction of residues is naturally selected for stability. Because the physical interactions underlying the selection of amino acids are very sensitive to the atomic details of the structure, the level of native amino acid recovery should be indicative of the accuracy of the protein structure. While redesigned template structures recovered only 23±6% of native amino acid sequences, redesigned X-ray and selected model structures recovered 35±10% and 42±8% of native amino acid sequences, respectively. Only 41±13% of the native residues recovered in redesigned templates were also recovered in redesigned X-ray structures. By contrast, 72±7% of the native residues recovered in redesigned selected models were also recovered in redesigned X-ray structures. These results indicate that the native interactions recovered in redesigned X-ray and selected model structures are similar, and suggest that the TMH regions of protein models generated using our method are in a range of accuracy suitable for rational design applications.

## Discussion

The prediction of membrane protein structures represents an important approach in light of their difficult experimental determination but remains a challenging problem. Current prediction techniques are limited to the generation of low-resolution models from sequence information alone [10]–[12] or of near-atomic resolution models from close structural homologues [5]. However, close structural homologs are currently not available for a large fraction of membrane proteins and often only one distant structural homolog hit can be found for these proteins (**Fig. S1**), making their structure prediction at high-resolution a real challenge. To address this problem, we developed a generic method that can efficiently reconstruct TMH and loop regions from single distant or closer homologues. The method was stringently validated in two blind predictions and in a large benchmark consisting of pairs of membrane protein homologues with wide diversity in length, topology and sequence identity. Submitted models were first-ranked in the blind predictions [14], [15] for the accuracy of the full-length receptor structure and the method was able to improve most starting templates in the benchmark to reach near atomic accuracy prediction in the TMH regions (**Fig. 2**, **Fig. 5**, **Table S1**). In local regions of the TMH structures where distortions differed between template and target, the method was able to significantly improve the starting template and to predict distorted helical structures with an average Cα RMSD of only 0.8 Å to the native structures (**Fig. 3** **, Table S2**). As a stringent proof of the model's accuracy, complete redesign of their TMH regions recapitulated similar native interactions than the redesign of the same regions in the X-ray structures. In contrast, the methods MEDELLER [30], a comparative modeling technique developed for membrane proteins, the widely used homology modeling software MODELLER [31], and I-TASSER, a web-server for protein structure prediction [32], [33], did not significantly improve homologous templates (**Fig. 5**).

The improvements observed for most distant or closer homologues with diverse length and topology indicate that the method provides a general and efficient approach for reconstructing the structure of a large diversity of transmembrane helical folds. Starting templates with sequence identity to the target as low as 15% were significantly improved, suggesting that the technique should be effective at generating atomic-level models more accurate than available templates for many structurally uncharacterized TMH proteins (**Fig. S1**).

Because the conformational heterogeneity and poor stability of eukaryotic membrane proteins in detergents is a major bottleneck to their crystallization, their stabilization has been a very intensive area of research but has only been achieved with limited success using labor-intensive cycles of random or scanning mutagenesis [36]–[38]. According to our design calculations, our technique can predict stabilizing physical interactions in structurally uncharacterized receptors and should therefore be particularly useful for predicting mutational effects on receptor's conformational stability, for engineering receptors with altered conformational energy landscape and for precisely guiding structure/function studies.

Future developments will involve 1) the explicit modeling of water molecules to improve the prediction of TMH core regions, and 2) the simultaneous modeling of loop and bound ligand conformations to improve the prediction of loop structures and allow accurate prediction of receptor-ligand bound conformations and interactions for ligand docking and virtual screening applications.

In conclusion, the method may prove useful for the atom-level modeling and design of structurally uncharacterized classes of alpha-helical membrane receptors which are particularly challenging to study experimentally and for which close homologues are currently often not available.

## Methods

### Identification of structural homologs for all human transmembrane helical proteins

To analyze the coverage potential of homology modeling of membrane proteins, HHpred [25], a toolkit for searching and aligning query sequences with sequences from existing structures, was run on three datasets of human transmembrane helical proteins. Two datasets were taken from the *Survey of the Human Transmembrane Proteome* [39] and consisted in: 1) full-length sequences of human transmembrane proteins with at least two predicted transmembrane helices (3838 sequences), and 2) full-length sequences of human transmembrane proteins with at least two predicted transmembrane helices truncated to the transmembrane domain (i.e. from the first to last predicted transmembrane helix residues) (3838 sequences). Additionally, a full-length human transmembrane proteome dataset (6521 sequences) was created by supplementing the aforementioned 3838 full-length multi-pass sequences with 2683 human single-pass transmembrane helical proteins from Uniprot database [40]. Each of these datasets were clustered at 98% sequence identity using USEARCH [41], yielding non-redundant dataset sets of 3405, 3079, and 5818 for the full-length human multi-pass transmembrane helical proteins, transmembrane domain truncated human multi-pass transmembrane helical proteins, and full-length combined single- and multi-pass human transmembrane helical proteins, respectively. These were used as inputs to HHpred search for structurally characterized homologs. HHpred was run by using the HHsuite programs HHblits [26] (to generate HMM alignment from searching Uniprot database) and HHsearch [27] (to match the HMM-HMM alignment to PDB database). DSSP [42] and Psipred [43] were used for secondary structure prediction annotation as part of the HHpred protocol. The resultant HHpred alignments were filtered by a range of percent sequence identity thresholds (i.e. of homolog hit versus query) and percent coverages (i.e. of total length of query sequence) of 90%, 75%, 60% or 50%.

### Dataset of membrane protein structures for the benchmark

A representative dataset of 50 membrane protein structure pairs was selected that samples a wide range of sequence identity (from 15% to 70%), length (from 183 to 420 residues) and topology (from 5 to 12 TMHs). As outlined below, the dataset was selected to be representative of the entire classes of membrane proteins that can be modeled using the method described in this study.

### Selection of modeling targets from the Orientation of Proteins in Membranes (OPM) database[44]

Membrane protein targets were selected by filtering the OPM database with the following criteria that reflect the current scope of the method. Firstly, selecting for “transmembrane” and “alpha-helical polytopic”, 936 proteins in 75 superfamilies were identified. Next, families were removed that 1) have less than two unique protein structures (need at least one homolog) –or– 2) consist of multi-protein complexes –or– 3) consist of very large proteins (>15 secondary structure elements or >600 residues) –or– 4) contain large cofactors (e.g. heme groups) –or– 5) formed from many symmetrical subunits. This reduced the number of superfamilies to 18. Additionally, four of the remaining superfamilies did not contain proteins with structurally characterized homologs with sequence identity >15% and were also removed. The remaining 14 superfamilies are the following (as categorized by OPM database): 1) Rhodopsin-like receptors and pumps, 2) ABC transporters, 3) General secretory pathway, 4) Major Intrinsic Protein, 5) Ammonia and urea transporters, 6) Major Facilitator Superfamily, 7) APC (Amino acid-Polyamine-organoCation) superfamily, 8) Monovalent cation-proton antiporter, 9) Chloride transporter, 10) Multidrug/Oligosaccharidyl-lipid superfamily, 11) Energy-coupling factor transporters, 12) Rhomboid protease, 13) Sodium/calcium exchanger, and 14) Peptidase family M48. Our dataset of modeling targets covers 12 of 14 superfamilies. The available target/template homologs for the Monovalent cation-proton antiporters and the Peptidase family M48 are too distant (structural alignment between template and target is extremely poor: Calpha rmsd = 25 Å) and too homologous (38% identity), respectively, to be considered relevant for this study. In total we selected 50 representative modeling cases combining different target/template pairs, and 31 unique targets. Of our modeling targets, 12 are GPCRs (11 Class A and 1 Class B) and 19 are non-GPCRs membrane proteins.

The following X-ray structures and corresponding pdb codes were selected from the protein database:

1. *GPCRs:* Bovine rhodopsin (1U19), Squid rhodopsin (2Z73), Beta2 adrenergic receptor (2RH1), Beta1 adrenergic receptor (2Y00), Adenosine A2A receptor (3EML), Dopamine D3 receptor (3PBL), Chemokine receptor CXCR4 (3ODU), Kappa opioid receptor (4DJH), M2 muscarinic acetylcholine receptor (3UON), Histamine H1 receptor (3RZE), Sphingosine 1-phosphate receptor 1 (3V2Y), Delta opioid receptor (4EJ4), M3 Muscarinic Acetylcholine Receptor (4DAJ), human glucagon receptor (4L6R), corticotropin-releasing factor receptor 1 (4K5Y).
2. *non-GPCRs:* Aquaporins (1J4N, 3GD8), Glycerol channel (1FX8), Formate channel (3KLY), Arginine antiporter (3L1L), Lactose Permease (2CFQ), EmrD multidrug transporter (2GPF), *E. coli* GlpG rhomboid family intramembrane protease (2IC8), GlpG, Rhomboid Peptidase from *Haemophilus influenzae* (2NR9), *E. coli* BtuCD protein, an ABC transporter mediating vitamin B12 uptake (1L7V), putative metal-chelate-type adenosine triphosphate (ATP)-binding cassette (ABC) transporter from *Haemophilus influenzae* (2NQ2), *E. coli* Ammonia Channel AmtB (1U7G), Ammonium Transporter Amt-1 from *Archaeoglobus fulgidus* (2B2H), Rhesus protein Rh50 from *Nitrosomonas europaea* (3B9W), ECF-type riboflavin transporter from *Staphylococcus aureus* (3P5N), thiamine-specific S-component ThiT ECF-type transporter from *Lactococcus lactis* (3RLB), apo-ApcT, a proton-coupled broad-specificity amino acid transporter (3GIA), Glu-GABA antiporter GadC, a member of the amino-acid-polyamine-organocation superfamily of membrane transporters (4DJK), Cyanobacterial Cl−/H+ antiporter (3ND0), eukaryotic CLC transporter (3ORG), Protein translocases SecY (1RH5, 2ZJS), Energy-coupling factor transporter EcfA (4HZU), Sodium/calcium exchanger (3V5U), Proton/calcium exchanger (4KPP), Proton-driven MATE exporter (3VVO), Sodium/drug antiporter NorM (3MKT), Rhesus Glycoprotein RhCG (3HD6).

### Sequence alignment between target and templates

Several methods including the consensus method 3D-Jury [45] and HHpred [25] based on HMM-HMM comparisons were tested to generate optimal sequence-sequence alignments. HHpred gave the best alignments in our benchmark and was subsequently used for all predictions. The following parameters were used: ten PSI-BLAST iterations with an E-value threshold of 1E-3, local alignment with global final realignment. For the blind predictions, the best alignment was systematically considered to select homologues and construct templates. For a few of the most distant pairs (3L1L from 3GIA, 2CFQ from 2GPF, 3P5N from 4DVE, 3GIA from 4DJK, 2GPF from 2CFQ, 3KLY from 3GD8, 3GD8 from 3KLY, 3HD6 from 1U7G, 3VVO from 3MKT and 4HZU from 3RLB), the sequence alignment generated by HHpred was adjusted manually, guided by topology prediction of TMHs given by Octopus [46] and secondary structure prediction given by Psipred [43], to improve the alignment of the TMH region and minimize the number gaps or insertions in this region.

### Generation of models using MEDELLER

The template structures and alignments between template and target sequences for each protein pair in the benchmark were used as inputs to the Homology Modeling software MEDELLER [30]. MEDELLER was run using the online MEDELLER server (http://opig.stats.ox.ac.uk/webapps/medeller/home.pl?app=MEDELLER) with default settings to generate “complete” models. The MEDELLER server does not provide a benchmarking option that excludes the target structure from its loop modeling process, which uses FREAD [39], a database search loop modeling algorithm. Therefore, all models generated by MEDELLER were checked for loops that incorporated fragments from the target structure. For all but two protein pairs in the benchmark, the complete models generated by the MEDELLER server did not include target loops and were directly used for analysis. The two MEDELLER models (3EML from 3UON and 1U7G from 3B9W) that included target loops were run again on the online FREAD server (http://opig.stats.ox.ac.uk/webapps/fread/php/index.php) and the best loop fragment hits excluding those from the target were used for analysis.

### Generation of models using MODELLER

Homology modeling with MODELLER [31] was run using an online MODELLER server (http://toolkit.tuebingen.mpg.de/modeller) with default settings. The template structures and alignments between template and target sequences for each protein pair in the benchmark were used as inputs.

### Generation of models using I-TASSER

The I-TASSER server (http://zhanglab.ccmb.med.umich.edu/I-TASSER/) was provided with the same target sequence, target/template alignment and template structure than Rosetta, MODELLER and MEDELLER (option I: Specify template with alignment). To ensure that I-TASSER would not use any additional homolog templates closer to the target than the one assigned in each protein pair of the benchmark, other templates with sequence identity higher than 25% to the target or closely related to the homolog template assigned in each protein pair were excluded (option II: Exclude homologous templates/Exclude specific template proteins). I-TASSER usually generated 5 models and the most accurate one is reported in our study.

### Rebuilding-and-Refinement protocol

The method consists of three parts: **1.** Rebuilding of TMH structures, **2.** Rebuilding of non-TMH (e.g. loops, helical bends) structures, **3.** All-atom refinement of reconstructed structures.

**1.** Rebuilding of TMH structures is performed if 1) gaps in the sequence alignment occur in these regions, 2) bends have different predicted positions (e.g. unaligned Prolines or coil motifs, non-conservation of Prolines between template and target sequences) or 3) TMHs have different predicted lengths (i.e. significantly different secondary structure prediction) indicating potential different tilt angles with regards to the membrane plane. Concerning the prediction of residues promoting helical bends, we limited ourselves to the presence of prolines in the target or in 10% of the homolog sequences which, depending on the membrane protein structure databases analyzed, account for between 60% [21] and 90% [18] of TMH kinks. Sequence motifs other than prolines have been reported to induce helical bends but current sequence-based predictions do not exhibit a combined sensivity/specificity high enough to be used as an automated input in the rebuilding of TMHs. Even if they cannot be identified by sequence or secondary structure information alone, helical bends and distortions promoted by local strain in the backbone structure or by specific tertiary interactions can still be identified and modeled during the all-atom structure refinement stage.

Rigid-body helical degrees of freedom of TMHs to be rebuilt are sampled based on a kinematic description of the polypeptide chain where the protein system is represented in internal coordinates by a tree of atoms which can have any structure provided there is no closed loop [10], [47]. The atom-tree representation was further developed so that the edges in the tree can be any bond connections or rigid body transformations, making the protein a single continuous bonded chain or multiple domains connected by virtual long-range “jumps” between residues. This new atom-tree representation allows torsional and rotameric sampling within each individual TMH segment as well as perturbations in the rigid body degrees of freedom around the “jump” connecting these segments. Loops and local distorted regions connecting full-length or fragments of TMHs to be rebuilt are stripped out from the template and alternative TMH conformations are generated by randomly sampling rigid body degrees of freedom along and off the helical axis. At this stage, the protein template is represented at the coarse-grained level where side-chain conformations are averaged out, thereby drastically decreasing the number of degrees of freedom to be sampled. Moves are accepted using a Metropolis Monte-Carlo criterion (1000 to 5000 steps for each TMH fragment constrained by a Gaussian function to 1–1.5 Å of the starting structure) and followed by loop rebuilding and full structure gradient-based minimization (see below).

More specifically, as shown in **Fig. 1**, kinked TMHs are represented by two TMH fragments and a distorted helical turn around the kink. Each helix fragment is treated as a rigid-body and defined by a helical axis (m for “moveable” and f for “fixed” defining the reference state). Following the distribution of kink angles and distances between TMH fragments of kinked TMHs in membrane protein structures, the two TMH fragments adopt relative orientations that are constrained in space. The m helix is moved with regard to the f helix according to the following degrees of freedom:

1. A translation is applied to the m helix moving its C-terminus to a random point on the surface of a hemisphere originating at the N-terminal of the fixed helix, with a gaussian radius equal to 7.2±0.6 Å (only +z-axis translation is allowed) [24].
2. A rotation is applied to the m helix. The rotation is to a random vector restricted to the +z, and +x quadrants (−x being defined as the vector between the m helix C-terminus and the Carbon Beta (CB) atom of the f helix N-terminal residue, e.g. proline). These moves ensure that the projection of the N-terminus of the m helix on a plane orthogonal to the f axis and crossing the proline ring preferentially occupies the semicircle away from the proline ring as observed in kinked TMH native structures (**Fig. 1**) [23].

In addition to these constrained moves, each TMH fragment is allowed to spin around its helical axis. Finally, the f and m helices are also allowed to move as a single unit and to sample the conformational degrees of freedom of a standard alpha helix rigid body.

**2.** Non-TMH (e.g. loops, helical bends) structures with low sequence identity to the template or exhibiting gaps/deletions in the sequence alignment with the template are rebuilt de novo [10]. This step follows the previously developed de novo folding protocol for membrane protein structures and involves random peptide fragment insertions subjected to acceptation by the Metropolis criteria based on the total energy of the system. At this stage, the system is still represented at the coarse-grained level and the low-resolution energy function of Rosetta is used to compute the energy of the system. Cyclic coordinate descent (CCD) is used to close the chain break in the rebuilt region and to maintain the connectivity of the protein chain, and is achieved by iteratively inserting fragments and increasing the chain break penalty. If after twelve rebuilding steps, any chain break remains larger than 0.2 Å, the region to be rebuilt is expanded by one residue on both sides until a continuous peptide chain is recovered. The libraries of fragments to be inserted are generated for fragments of size 9 and 3. Fragments of larger size were tested but didn't provide any significant improvements in the accuracy of the rebuilt regions.

Helical bends in kinked TMHs are typically modeled as four residues loop insertion connecting two helical fragments and can sometime result in distorted loop conformations which are not usually observed in native kinked helices. Such local structures involve either a combination of non-helical turns and 3_10_ helix or helical distortions extending 2 or 3 residues Cterminal to the residue responsible for the bend. In such situation, starting from the selected all-atom refined model, a larger window of residue (e.g. 5 to 8) is rebuilt and locally refined using the loop modeling protocol.

**3.** The fragment insertion protocol described above involves fragment insertion moves that sample a large conformational space to identify a broad range of physically-realistic conformations. The coarse-grained models are then subjected to all-atom refinement which searches the all-atom conformational energy landscape for local minima in the vicinity of these structures. This step combines an all-atom energy function developed for transmembrane protein structures with an efficient search for low-energy conformations. As described previously [24], the energy function mainly consists of short-range interactions, e.g. Lennard-Jones, hydrogen-bond. Knowledge-based potentials describe torsional states of both backbone and side-chain atoms and the solvation energy of each atom as a function of both its depth in the membrane and its burial in the protein. A Monte-Carlo minimization procedure with discrete side-chain optimization is used to efficiently sample low-energy conformations in the rugged all-atom energy landscape. A single move involves the following steps: 1) random backbone perturbations, 2) discrete side-chain optimization for the new backbone conformation, 3) minimization of the energy of the system with respect to all conformational degrees of freedom. Several cycles of small backbone perturbations are first applied to the entire receptor starting with a smooth Lennard-Jones potential followed by an iterative ramping up of the repulsive part of the potential. This procedure allows a smooth transition from a coarse-grained to a full-atom representation without loosing the compactness of the initial structures.

To avoid the sampling of conformational space unlikely occupied by the target sequence, the all-atom energy function is also supplemented by a constraint potential maintaining conserved regions that are in vicinity in the template structures. These constraints are defined between pairs of strictly conserved or similar residues in both target and template sequences and that are in vicinity in the template structures. In our calculations, a constraint is defined by a distance between the Cα atoms of the interacting residue pairs and a constraint width (i.e. the deviation from the assigned distance at which the constraint score begins to ramp up). Any deviation from these distances during refinement is penalized by a harmonic potential. Small constraint widths were assigned for short-range contacts (e.g. 0.2 Å for contacts ≤5 Å) while larger constraint widths were assigned for longer-range constraints (e.g. 0.5 Å for contacts of ∼8 Å). To avoid over constraining the models to the starting template, the average number of selected constraints was around 5% of the total number of residues for the most distant homolog pairs (i.e. sequence identity of 15–20%) and between 5% and 10% for intermediate homolog pairs (i.e. sequence identity of 20–25%). For closer homolog pairs (i.e. sequence identity >25%), models were highly constrained to the starting templates at most positions not rebuild *de novo*.

### Selection of models

Between 10000 and 40000 all-atom refined models are generated per target. 1000 or up to 10% lowest energy structures are selected and their transmembrane region clustered into structurally-related families using Rosetta's clustering protocol. The most accurate model among the five lowest-energy structures which cluster in one of the five largest families of models is selected and discussed in this study. Accuracy of the models to the target structure is computed using TM-SCORE [48] over full length and TM regions.

### Sequence design calculations

Sequence design calculations were performed as described previously [24], [49]. Briefly, the backbone coordinates from the X-ray structure, the selected model and the initial template were selected to perform the design calculations. All 20 amino acids were allowed at the TMH positions and the native residues were kept in the loop regions. The combination of amino acids and side-chain conformations minimizing the free energy of the system was selected by Monte Carlo sampling of discrete side-chain conformations (i.e. rotamers) followed by energy minimization over all conformational degrees of freedom. The Dunbrack rotamer library [50] expanded by rotamers at +−1 standard deviation around the mean values for the dihedral angles chi1 and chi2 was used to repack the structures. The energy of each structure was computed using the all-atom RosettaMembrane energy function [24]. 100 independent design calculations were performed starting from each individual backbone structure. The percentage of native sequence recovery was calculated from the lowest energy designed structures.

## Supporting Information

Figure S1 **Homology modeling coverage for the human multi-pass TMH proteome.** **A.** Percent of hits (i.e. structural homologs) as calculated by HHpred [25]–[27] for all full-length human multi-pass TMH proteins (3405 annotated sequences [39]) split in three target/template sequence identity thresholds: distant (percent sequence identity between target and template between 15 and 25%: %ID 15–25), medium (%ID 25–35) and close homology (%ID >35) thresholds. The data is represented for four levels of target sequence length coverage by the template: 50% (green), 60% (red), 75% (grey) and 90% (blue). **B.** Distribution of hits in the distant homology (%ID 15–25) bin for all full-length human multi-pass TMH proteins. The fraction of transmembrane proteins for which 1, 2, 3, 4 or more than 4 distant homolog templates were identified by HHpred is represented for 75% target sequence length coverage by the template. **C, D.** Percent of hits (i.e. structural homologs) as calculated by HHpred [25]–[27] for all full-length human multi-pass and single-pass TMH proteins (5818 annotated sequences, **C**) or for all TM domains (i.e. from the first to the last TMH residue) of all human multi-pass TMH proteins (3079 annotated sequences [39], **D**), split in three target/template sequence identity thresholds: distant (percent sequence identity between target and template between 15 and 25%: %ID 15–25), medium (%ID 25–35) and close homology (%ID >35) thresholds. The data is represented for four levels of target sequence length coverage by the template: 50% (green), 60% (red), 75% (grey) and 90% (blue).
(DOCX)

Table S1 **Improvement of model accuracy.** The most accurate among the five lowest energy selected Rosetta models (see Methods) is reported in the table. If the selected model does not belong to the lowest energy cluster, the Cα rmsd of the lowest energy model from the lowest energy cluster is also reported in parentheses next to that of the selected model for the TMH region. For comparison, the most accurate among five models generated by the methods Modeller, Medeller and I-TASSER is reported. ^a^ Sequence identity between target and template sequences calculated by HHpred [25] over aligned full length or modeled regions. ^b^ Mode of Rosetta used to generate models: TMH rebuilding mode (RBK), Regular loop relax (LR). ^c^ R.m.s. deviation over Cα atoms (in Å) to the crystal structure. ^d^ Geometric Distance Test with High-Accuracy [34]. This value is the average of four-numbers: the numbers of residues aligned between template or model and crystal structure within 0.5 Å, 1 Å, 2 Å and 4 Å [34]. ^e^ Percentage of residues superimposable within 2 angstroms of the crystal structure. ^f^ Percentage of residues superimposable within 1 angstrom of the crystal structure. ^g^ Transmembrane helical (TMH) region is defined by the helices spanning the lipid membrane.
(DOCX)

Table S2 **Improvement of model accuracy in the distorted second TMH of GPCRs.** The most accurate among the five lowest energy selected models (see Methods) is reported in the table. ^a^ R.m.s. deviation over Cα atoms (in Å) of TMH2 to the crystal structure. ^b^ Geometric Distance Test (GDT). This value is the average of four-numbers: the numbers of residues aligned between template or model and crystal structure within 1 Å, 2 Å, 4 Å and 8 Å [48]. ^c^ Geometric Distance Test with High-Accuracy (GDT-HA). This value is the average of four-numbers: the numbers of residues aligned between template or model and crystal structure within 0.5 Å, 1 Å, 2 Å and 4 Å [34]. <ΔPHI> and <ΔPSI> represents the average deviation of backbone dihedral angles between template or model and native structure in the *de novo* rebuilt bend region.
(DOCX)
