# Supplementary material for: High-Resolution Modeling of Transmembrane Helical Protein Structures from Distant Homologues
Source: PLoS Comput Biol. 2014 May 22;10(5):e1003636. doi: 10.1371/journal.pcbi.1003636 (PMC4031050; doi:10.1371/journal.pcbi.1003636)
Supplement: Figure S1 — Homology modeling coverage for the human multi-pass TMH proteome. A. Percent of hits (i.e. structural homologs) as calculated by HHpred [25]–[27] for all full-length human multi-pass TMH proteins (3405 annotated sequences [39]) split in three target/template sequence identity thresholds: distant (percent sequence identity between target and template between 15 and 25%: %ID 15–25), medium (%ID 25–35) and close homology (%ID >35) thresholds. The data is represented for four levels of target sequence length coverage by the template: 50% (green), 60% (red), 75% (grey) and 90% (blue). B. Distribution of hits in the distant homology (%ID 15–25) bin for all full-length human multi-pass TMH proteins. The fraction of transmembrane proteins for which 1, 2, 3, 4 or more than 4 distant homolog templates were identified by HHpred is represented for 75% target sequence length coverage by the template. C, D. Percent of hits (i.e. structural homologs) as calculated by HHpred [25]–[27] for all full-length human multi-pass and single-pass TMH proteins (5818 annotated sequences, C) or for all TM domains (i.e. from the first to the last TMH residue) of all human multi-pass TMH proteins (3079 annotated sequences [39], D), split in three target/template sequence identity thresholds: distant (percent sequence identity between target and template between 15 and 25%: %ID 15–25), medium (%ID 25–35) and close homology (%ID >35) thresholds. The data is represented for four levels of target sequence length coverage by the template: 50% (green), 60% (red), 75% (grey) and 90% (blue). (DOCX) [file pcbi.1003636.s001.docx]

**Supporting Information**

**Chen, K.M., Sun, J., Salvo, J., Baker, D., Barth, P.**

**Supplementary Figure 1. Homology modeling coverage for the human multi-pass TMH proteome.** **A.** Percent of hits (i.e. structural homologs) as calculated by HHpred [^1^](#_ENREF_1)^,^ [^4^](#_ENREF_4)^,^ [^5^](#_ENREF_5) for all full-length human multi-pass TMH proteins (3405 annotated sequences [^6^](#_ENREF_6)) split in three target/template sequence identity thresholds: distant (percent sequence identity between target and template between 15 and 25%: %ID 15-25), medium (%ID 25-35) and close homology (%ID >35) thresholds. The data is represented for four levels of target sequence length coverage by the template: 50% (green), 60% (red), 75% (grey) and 90% (blue). **B.** Distribution of hits in the distant homology (%ID 15-25) bin for all full-length human multi-pass TMH proteins. The fraction of transmembrane proteins for which 1, 2, 3, 4 or more than 4 distant homolog templates were identified by HHpred is represented for 75% target sequence length coverage by the template. **C, D.** Percent of hits (i.e. structural homologs) as calculated by HHpred [^1^](#_ENREF_1)^,^ [^4^](#_ENREF_4)^,^ [^5^](#_ENREF_5) for all full-length human multi-pass and single-pass TMH proteins (5818 annotated sequences, **C**) or for all TM domains (i.e. from the first to the last TMH residue) of all human multi-pass TMH proteins (3079 annotated sequences [^6^](#_ENREF_6), **D**), split in three target/template sequence identity thresholds: distant (percent sequence identity between target and template between 15 and 25%: %ID 15-25), medium (%ID 25-35) and close homology (%ID >35) thresholds. The data is represented for four levels of target sequence length coverage by the template: 50% (green), 60% (red), 75% (grey) and 90% (blue).

**
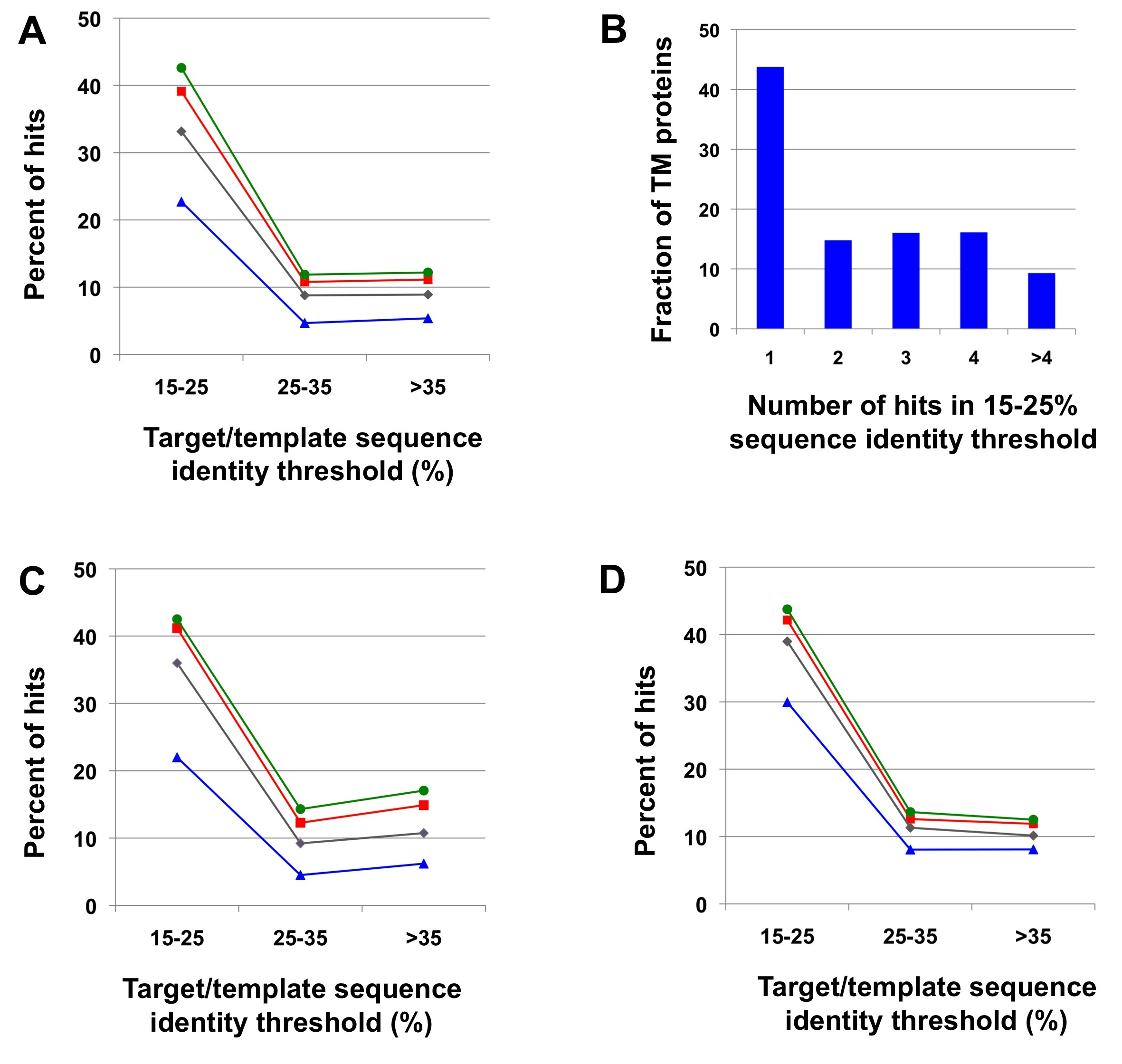
**
