# Supplementary material for: High-Resolution Modeling of Transmembrane Helical Protein Structures from Distant Homologues
Source: PLoS Comput Biol. 2014 May 22;10(5):e1003636. doi: 10.1371/journal.pcbi.1003636 (PMC4031050; doi:10.1371/journal.pcbi.1003636)
Supplement: Table S1 — Improvement of model accuracy. The most accurate among the five lowest energy selected Rosetta models (see Methods) is reported in the table. If the selected model does not belong to the lowest energy cluster, the Cα rmsd of the lowest energy model from the lowest energy cluster is also reported in parentheses next to that of the selected model for the TMH region. For comparison, the most accurate among five models generated by the methods Modeller, Medeller and I-TASSER is reported. a Sequence identity between target and template sequences calculated by HHpred [25] over aligned full length or modeled regions. b Mode of Rosetta used to generate models: TMH rebuilding mode (RBK), Regular loop relax (LR). c R.m.s. deviation over Cα atoms (in Å) to the crystal structure. d Geometric Distance Test with High-Accuracy [34]. This value is the average of four-numbers: the numbers of residues aligned between template or model and crystal structure within 0.5 Å, 1 Å, 2 Å and 4 Å [34]. e Percentage of residues superimposable within 2 angstroms of the crystal structure. f Percentage of residues superimposable within 1 angstrom of the crystal structure. g Transmembrane helical (TMH) region is defined by the helices spanning the lipid membrane. (DOCX) [file pcbi.1003636.s002.docx]

**Supporting Information**

**Chen, K.M., Sun, J., Salvo, J., Baker, D., Barth, P.**

**Supplementary Table 1. Improvement of model accuracy.** The most accurate among the five lowest energy selected Rosetta models (see Methods) is reported in the table. If the selected model does not belong to the lowest energy cluster, the Cα rmsd of the lowest energy model from the lowest energy cluster is also reported in parentheses next to that of the selected model for the TMH region. For comparison, the most accurate among five models generated by the methods Modeller, Medeller and I-TASSER is reported. ^a^ Sequence identity between target and template sequences calculated by HHpred [^1^](#_ENREF_1) over aligned full length or modeled regions. ^b^ Mode of Rosetta used to generate models: TMH rebuilding mode (RBK), Regular loop relax (LR). ^c^ R.m.s. deviation over Cα atoms (in Å) to the crystal structure. ^d^ Geometric Distance Test with High-Accuracy [^2^](#_ENREF_2). This value is the average of four-numbers: the numbers of residues aligned between template or model and crystal structure within 0.5 Å, 1 Å, 2 Å and 4 Å [^2^](#_ENREF_2). ^e^ Percentage of residues superimposable within 2 angstroms of the crystal structure. ^f^ Percentage of residues superimposable within 1 angstrom of the crystal structure. ^g^ Transmembrane helical (TMH) region is defined by the helices spanning the lipid membrane.

| X-ray structure | Receptor template | Sequence identity to template^a^ (modeled / full length input, %) | Rosetta mode^b^ | Full length | | | | | TMH region^g^ | | | | |
| --- | --- | --- | --- | --- | --- | --- | --- | --- | --- | --- | --- | --- | --- |
|  |  |  |  | (Cα rmsd (Å)^c^ / GDT-HA^d^) | | | | | (Cα rmsd (Å)^c^ / GDT-HA^d^) | | | | |
|  |  |  |  | (<2 Å^e^ / <1 Å^f^) | | | | | (<2 Å^e^ / <1 Å^f^) | | | | |
|  |  |  |  | Template | Modeller | Medeller | I-TASSER | Rosetta | Template | Modeller | Medeller | I-TASSER | Rosetta |
| 1U19 | 3EML | 20 / 17 | LR | 6.2 / 0.40 | 6.3 / 0.39 | 7.7 / 0.42 | 6.0 / 0.41 | 5.1 / 0.48 | 1.8 / 0.54 | 1.8 / 0.55 | 1.7 / 0.57 | 1.7 / 0.55 | 1.3 / 0.70 |
|  |  |  |  | 56 / 18 | 55 / 17 | 60 / 19 | 58 / 20 | 64 / 33 | 77 / 28 | 78 / 28 | 81 / 31 | 80 / 31 | 94 / 60 |
| 1U19 | 2Z73 | 28 / 24 | LR | 3.1 / 0.54 | 2.9 / 0.53 | 3.0 / 0.54 | 3.0 / 0.51 | 2.1 / 0.64 | 1.5 / 0.61 | 1.5 / 0.61 | 1.4 / 0.61 | 1.6 / 0.58 | 1.0 / 0.74 |
|  |  |  |  | 80 / 33 | 80 / 31 | 80 / 33 | 76 / 23 | 89 / 55 | 92 / 41 | 91 / 40 | 92 / 41 | 87 / 32 | 99 / 70 |
| 1U19 | 3ODU | 19 / 19 | RBK | 5.8 / 0.39 | 5.8 / 0.39 | 5.5 / 0.42 | 3.9 / 0.46 | 4 / 0.54 | 2.0 / 0.55 | 2.0 / 0.56 | 2.0 / 0.56 | 2.1 / 0.52 | 1.3 (1.2) / 0.70 |
|  |  |  |  | 49 / 23 | 49 / 23 | 53 / 24 | 62 / 26 | 72 / 47 | 72 / 34 | 73 / 35 | 72 / 36 | 71 / 29 | 92 / 66 |
| 2RH1 | 1U19 | 17 / 18 | LR | 5.0 / 0.42 | 5.0 / 0.42 | 5.2 / 0.42 | 4.8 / 0.40 | 4.2 / 0.55 | 2.0 / 0.54 | 2.0 / 0.54 | 1.9 / 0.54 | 2.1 / 0.52 | 1.4 / 0.73 |
|  |  |  |  | 59 / 18 | 60 / 18 | 59 / 18 | 52 / 17 | 70 / 46 | 76 / 28 | 76 / 29 | 76 / 28 | 71 / 29 | 91 / 73 |
| 2RH1 | 2Z73 | 23 / 20 | LR | 4.6 / 0.46 | 4.5 / 0.46 | 4.9 / 0.45 | 3.8 / 0.45 | 4.0 / 0.53 | 1.8 / 0.60 | 1.7 / 0.60 | 1.8 / 0.61 | 1.9 / 0.59 | 1.3 (1.7) / 0.72 |
|  |  |  |  | 61 / 30 | 61 / 30 | 60 / 30 | 57 / 30 | 65 / 41 | 81 / 46 | 78 / 46 | 80 / 47 | 74 / 45 | 90 / 67 |
| 2RH1 | 3ODU | 21 / 20 | RBK | 6.1 / 0.38 | 5.6 / 0.37 | 5.8 / 0.40 | 6.2 / 0.36 | 3.8 / 0.49 | 2.4 / 0.52 | 2.7 / 0.52 | 2.4 / 0.54 | 2.5 / 0.49 | 1.7 / 0.68 |
|  |  |  |  | 51 / 20 | 48 / 20 | 52 / 24 | 44 / 20 | 62 /40 | 71 / 31 | 69 / 30 | 71 / 36 | 61 / 28 | 86 / 64 |
| 3EML | 1U19 | 20 / 20 | RBK | 5.8 / 0.40 | 5.7 / 0.40 | 5.7 / 0.42 | 5.5 / 0.44 | 4.4 / 0.42 | 1.8 / 0.53 | 1.8 / 0.54 | 1.8 / 0.53 | 1.8 / 0.59 | 1.8 / 0.63 |
|  |  |  |  | 57 / 18 | 57 / 19 | 60 / 20 | 60 / 26 | 56 / 23 | 76 / 27 | 76 / 28 | 75 / 27 | 83 / 38 | 82 / 50 |
| 3EML | 2Y00 | 26 / 30 | LR | 3.7 / 0.51 | 4.8 / 0.50 | 4.8 / 0.52 | 4.0 / 0.50 | 4.1 / 0.52 | 2.3 / 0.68 | 1.6 / 0.67 | 2.3 / 0.68 | 2.3 / 0.67 | 1.6 (1.6) / 0.76 |
|  |  |  |  | 64 / 38 | 65 / 37 | 68 / 42 | 62 / 36 | 68 / 42 | 86 / 57 | 86 / 55 | 85 / 59 | 84 / 56 | 94 / 75 |
| 3EML | 2Z73 | 18 / 18 | RBK | 5.3 / 0.47 | 5.2 / 0.48 | 5.1 / 0.48 | 5.1 / 0.41 | 4.9 / 0.48 | 1.6 / 0.68 | 1.6 / 0.68 | 1.6 / 0.68 | 1.7 / 0.59 | 1.4 / 0.71 |
|  |  |  |  | 61 / 37 | 60 / 37 | 62 / 38 | 56 / 23 | 60 / 37 | 84 / 62 | 84 / 62 | 84 / 62 | 82 / 41 | 91 / 63 |
| 3EML | 3ODU | 18 / 17 | RBK | 6.0 / 0.36 | 7.4 / 0.34 | 6.5 / 0.36 | 5.7 / 0.39 | 4.6 / 0.45 | 2.1 / 0.53 | 3.6 / 0.49 | 2.7 / 0.51 | 1.8 / 0.56 | 1.5 / 0.64 |
|  |  |  |  | 44 / 19 | 44 / 18 | 47 / 18 | 51 / 21 | 63 / 28 | 70 / 32 | 66 / 29 | 69 / 30 | 75 / 37 | 89 / 49 |
| 2Z73 | 1U19 | 27 / 25 | RBK | 3.0 / 0.54 | 2.9 / 0.54 | 3.0 / 0.54 | 2.9 / 0.52 | 3.3 / 0.59 | 1.4 / 0.61 | 1.4 / 0.61 | 1.4 / 0.61 | 1.4 / 0.60 | 1.1 (1.4) / 0.72 |
|  |  |  |  | 80 / 33 | 80 / 33 | 79 / 33 | 77 / 31 | 82 / 47 | 92 / 39 | 91 / 38 | 92 / 38 | 88 / 38 | 97 / 67 |
| 2Z73 | 3EML | 18 / 17 | RBK | 5.5 / 0.48 | 5.5 / 0.46 | 6.1 / 0.49 | 5.6 / 0.47 | 3.5 / 0.51 | 1.7 / 0.68 | 1.5 / 0.66 | 1.5 / 0.68 | 2.1 / 0.68 | 1.3 / 0.71 |
|  |  |  |  | 61 / 37 | 60 / 34 | 61 / 39 | 63 / 37 | 66 / 36 | 85 / 62 | 84 / 57 | 85 / 61 | 89 / 61 | 95 / 59 |
| 2Z73 | 3ODU | 20 / 18 | LR | 5.1 / 0.41 | 5.2 / 0.39 | 4.9 / 0.44 | 5.1 / 0.41 | 3.2 / 0.49 | 1.8 / 0.56 | 1.8 / 0.57 | 1.8 / 0.58 | 1.6 / 0.60 | 1.3 / 0.69 |
|  |  |  |  | 52 / 21 | 51 / 20 | 57 / 26 | 54 / 26 | 63 / 35 | 78 / 34 | 80 / 35 | 80 / 36 | 83 / 41 | 92 / 59 |
| 3PBL | 1U19 | 27 / 24 | LR | 5.4 / 0.48 | 3.7 / 0.45 | 4.0 / 0.46 | 3.6 / 0.48 | 2.9 / 0.53 | 1.4 / 0.60 | 1.5 / 0.60 | 1.4 / 0.60 | 1.4 / 0.62 | 1.1 / 0.73 |
|  |  |  |  | 67 / 28 | 63 / 24 | 65 / 25 | 66 / 30 | 68 / 42 | 89 / 38 | 85 / 38 | 90 / 38 | 87 / 46 | 94 / 71 |
| 3PBL | 3EML | 32 / 23 | LR | 2.6 / 0.59 | 2.5 / 0.59 | 2.5 / 0.61 | 2.6 / 0.56 | 2.2 / 0.60 | 1.1 / 0.76 | 1.1 / 0.74 | 1.1 / 0.75 | 1.1 / 0.70 | 1.1 / 0.76 |
|  |  |  |  | 74 / 48 | 75 / 48 | 79 / 51 | 75 / 44 | 74 / 48 | 97 / 72 | 97 / 69 | 97 / 70 | 99 / 62 | 97 / 73 |
| 3PBL | 3ODU | 21 / 20 | RBK | 5.2 / 0.45 | 4.9 / 0.46 | 4.8 / 0.47 | 5.2 / 0.45 | 3.4 / 0.54 | 1.7 / 0.61 | 1.7 / 0.61 | 1.7 / 0.59 | 1.7 / 0.59 | 1.2 / 0.74 |
|  |  |  |  | 60 / 27 | 61 / 28 | 61 / 27 | 60 / 27 | 68 / 44 | 85 / 44 | 85 / 44 | 84 / 41 | 85 / 38 | 94 / 71 |
| 3ODU | 2RH1 | 21 / 19 | RBK/LR | 6.2 / 0.41 | 6.1 / 0.42 | 5.1 / 0.42 | 5.9 / 0.40 | 5.8 / 0.43 | 2.8 / 0.52 | 2.7 / 0.52 | 2.8 / 0.53 | 3.0 / 0.50 | 1.9 / 0.58 |
|  |  |  |  | 52 / 24 | 52 / 27 | 54 / 26 | 46 / 27 | 55 / 28 | 68 / 34 | 68 / 35 | 68 / 35 | 90 / 58 | 75 / 42 |
| 3ODU | 2Z73 | 20 / 17 | RBK | 5.5 / 0.41 | 5.4 / 0.41 | 5.5 / 0.40 | 5.4 / 0.38 | 5.9 / 0.42 | 1.9 / 0.55 | 1.9 / 0.55 | 1.8 / 0.56 | 2.2 / 0.53 | 2.0 / 0.63 |
|  |  |  |  | 53 /22 | 53 / 23 | 51 / 22 | 45 / 21 | 53 / 28 | 77 / 34 | 77 / 33 | 79 / 34 | 67 / 34 | 78 / 54 |
| 3ODU | 3EML | 18 / 17 | RBK | 5.8 / 0.36 | 5.7 / 0.35 | 7.2 / 0.36 | 5.6 / 0.36 | 5.9 / 0.38 | 2.1 / 0.53 | 2.1 / 0.52 | 2.1 / 0.53 | 2.2 / 0.54 | 1.9 / 0.59 |
|  |  |  |  | 45 / 19 | 44 / 18 | 46 / 20 | 44 / 20 | 47 / 25 | 71 / 31 | 71 / 31 | 72 / 31 | 71 / 37 | 78 / 45 |
| 1J4N | 1FX8 | 30 / 27 | LR | 2.8 / 0.56 | 2.3 / 0.57 | 3.2 / 0.56 | 2.7 / 0.53 | 2.4 / 0.68 | 1.3 / 0.65 | 1.3 / 0.67 | 1.3 / 0.67 | 1.4 / 0.65 | 0.8 / 0.85 |
|  |  |  |  | 78 /37 | 78 / 38 | 75 / 38 | 79 / 33 | 84 / 62 | 93 / 47 | 95 / 52 | 93 / 51 | 92 / 48 | 99 / 85 |
| 1FX8 | 1J4N | 30 / 27 | LR | 2.8 / 0.56 | 2.6 / 0.57 | 2.5 / 0.56 | 2.7 / 0.57 | 2.2 / 0.63 | 1.3 / 0.66 | 1.3 / 0.66 | 1.3 / 0.66 | 1.2 / 0.70 | 1.2 (1.0) / 0.76 |
|  |  |  |  | 77 / 37 | 77 / 38 | 76 / 37 | 77 / 41 | 75 / 52 | 94 / 49 | 94 / 48 | 94 / 49 | 96 / 60 | 94 / 69 |
| 3KLY | 3GD8 | 18 / 17 | LR | 5.2 / 0.28 | 20.6 / 0.04 | 5.3 / 0.29 | 4.9 / 0.36 | 3.7 / 0.37 | 3.1 / 0.42 | 15.9 / 0.07 | 2.7 / 0.45 | 2.5 / 0.53 | 2.5 / 0.52 |
|  |  |  |  | 29 / 12 | 3 / 3 | 32 / 13 | 42 / 18 | 42 / 19 | 45 / 25 | 6 / 5 | 52 / 26 | 70 / 31 | 57 / 37 |
| 3L1L | 3GIA | 19 / 18 | LR | 4.1 / 0.33 | 4.1 / 0.35 | 4.2 / 0.34 | 4.0 / 0.35 | 3.7 / 0.40 | 2.8 / 0.43 | 2.7 / 0.43 | 2.8 / 0.42 | 2.6 / 0.43 | 2.0 (2.3) / 0.52 |
|  |  |  |  | 37 /16 | 40 / 24 | 39 / 17 | 40 / 17 | 55 / 18 | 48 / 23 | 49 / 24 | 47 / 23 | 51 / 21 | 76 / 27 |
| 2CFQ | 2GFP | 15 / 12 | RBK | 4.4 / 0.27 | 4.4 / 0.27 | 4.5 / 0.26 | 4.2 / 0.28 | 4.3 / 0.39 | 3.8 / 0.33 | 3.8 / 0.33 | 3.6 / 0.32 | 3.8 / 0.33 | 3.7 / 0.47 |
|  |  |  |  | 25 / 11 | 25 / 10 | 24 / 11 | 22 / 13 | 49 / 18 | 35 / 13 | 35 / 13 | 33 / 13 | 30 / 17 | 59 / 29 |
| 4DJH | 3UON | 25 / 24 | RBK | 3.8 / 0.48 | 3.8 / 0.48 | 3.9 / 0.48 | 3.7 / 0.49 | 3.0 / 0.51 | 1.7 / 0.64 | 1.7 / 0.65 | 1.7 / 0.63 | 1.6 / 0.64 | 1.6 / 0.72 |
|  |  |  |  | 64 / 31 | 64 / 31 | 64 / 31 | 65 / 32 | 64 / 38 | 84 / 51 | 85 / 55 | 85 / 48 | 84 / 52 | 87 / 62 |
| 3V2Y | 1U19 | 18 / 18 | RBK | 4.2 / 0.43 | 4.1 / 0.42 | 3.9 / 0.44 | 3.8 / 0.43 | 3.8 / 0.48 | 2.1 / 0.53 | 2.1 / 0.52 | 2.1 / 0.54 | 2.0 / 0.55 | 1.7 / 0.63 |
|  |  |  |  | 56 / 22 | 55 / 20 | 57 / 22 | 56 / 22 | 61 / 33 | 70 / 30 | 69 / 28 | 74 / 29 | 72 / 34 | 83 / 52 |
| 3V2Y | 3ODU | 15 / 16 | RBK | 4.7 / 0.39 | 4.6 / 0.38 | 4.3 / 0.38 | 4.5 / 0.37 | 3.9 / 0.46 | 2.3 / 0.50 | 2.4 / 0.50 | 2.4 / 0.51 | 2.3 / 0.49 | 1.8 (2.1) / 0.63 |
|  |  |  |  | 48 / 22 | 47 /21 | 47 / 23 | 46 / 19 | 60 / 33 | 67 / 32 | 65 / 32 | 67 / 35 | 66 / 26 | 84 / 50 |
| 3V2Y | 3RZE | 24 / 22 | RBK | 3.8 / 0.49 | 3.6 / 0.48 | 3.9 / 0.47 | 3.6 / 0.49 | 3.8 / 0.56 | 1.6 / 0.65 | 1.6 / 0.65 | 1.5 / 0.65 | 1.6 / 0.62 | 1.2 / 0.79 |
|  |  |  |  | 63 / 34 | 62 / 35 | 61 / 31 | 62 / 32 | 66 /49 | 84 / 55 | 85 / 57 | 84 / 58 | 84 / 48 | 94 / 77 |
| 4EJ4 | 3RZE | 26 / 26 | RBK | 2.8 / 0.55 | 2.8 / 0.55 | 3.0 / 0.51 | 2.8 / 0.54 | 2.6 / 0.58 | 1.5 / 0.67 | 1.4 / 0.67 | 1.9 / 0.62 | 1.5 / 0.64 | 1.5 (1.6) / 0.69 |
|  |  |  |  | 75 / 40 | 76 / 40 | 66 / 34 | 74 / 40 | 72 / 49 | 91 / 56 | 91 / 56 | 82 / 48 | 90 / 50 | 87 / 61 |
| 3UON | 3ODU | 16 / 15 | RBK | 4.0 / 0.42 | 3.8 / 0.43 | 3.6 / 0.45 | 4.0 / 0.44 | 3.8 / 0.49 | 2.0 / 0.56 | 2.0 / 0.56 | 1.9 / 0.58 | 2.0 / 0.57 | 1.7 / 0.64 |
|  |  |  |  | 56 / 24 | 55 / 24 | 60 / 28 | 60 / 26 | 66 / 34 | 77 / 39 | 75 / 37 | 78 / 42 | 78 / 41 | 88 / 49 |
| 4DAJ | 3ODU | 17 / 16 | LR | 4.1 / 0.42 | 4.0 / 0.43 | 4.1 / 0.43 | 3.9 / 0.43 | 3.3 / 0.47 | 2.0 / 0.57 | 1.9 / 0.59 | 2.0 / 0.58 | 1.8 / 0.59 | 1.5 / 0.65 |
|  |  |  |  | 53 / 27 | 53 / 27 | 54 / 27 | 55 / 25 | 60 / 30 | 75 / 44 | 76 / 46 | 76 / 44 | 80 / 44 | 90 / 53 |
| 4DAJ | 3UON | 64 / 45 | LR | 1.2 / 0.78 | 1.2 / 0.78 | 1.2 / 0.78 | 1.3 / 0.71 | 1.2 / 0.78 | 0.8 / 0.84 | 0.8 / 0.84 | 0.8 / 0.84 | 0.8 / 0.81 | 0.6 (0.6) / 0.89 |
|  |  |  |  | 93 / 77 | 93 / 78 | 93 / 78 | 92 / 69 | 95 / 79 | 99 / 89 | 100 / 89 | 99 / 89 | 99 / 87 | 100 / 98 |
| 3EML | 3UON | 24 / 24 | LR | 3.6 / 0.48 | 3.7 / 0.47 | 3.8 / 0.48 | 3.6 / 0.45 | 3.8 / 0.57 | 1.9 / 0.59 | 1.9 / 0.60 | 1.9 / 0.60 | 1.9 / 0.58 | 1.5 / 0.74 |
|  |  |  |  | 58 / 34 | 57 / 33 | 61 / 32 | 61 / 23 | 72 / 53 | 79 / 44 | 82 / 42 | 82 / 45 | 84 / 36 | 92 / 74 |
| 2IC8 | 2NR9 | 39 / 37 | RBK | 1.4 / 0.75 | 1.4 / 0.75 | 1.5 / 0.73 | 1.6 / 0.63 | 1.3 / 0.78 | 1.4 / 0.83 | 1.4 / 0.82 | 1.4 / 0.81 | 1.5 / 0.71 | 1.4 (1.6) / 0.84 |
|  |  |  |  | 91 / 74 | 91 / 75 | 89 / 72 | 87 / 51 | 94 / 81 | 93 / 87 | 93 / 85 | 92 / 85 | 90 / 69 | 93 / 89 |
| 1L7V | 2NQ2 | 32 / 32 | RBK | 2.9 / 0.50 | 2.8 / 0.50 | 2.7 / 0.51 | 2.9 / 0.48 | 2.8 / 0.57 | 2.2 / 0.60 | 2.2 / 0.61 | 2.2 / 0.60 | 2.4 / 0.58 | 1.1 (1.9) / 0.73 |
|  |  |  |  | 62 / 37 | 63 / 37 | 65 / 35 | 60 / 34 | 74 / 46 | 72 / 49 | 73 / 51 | 72 / 49 | 68 / 46 | 95 / 69 |
| 1U7G | 2B2H | 38 / 39 | RBK | 2.2 / 0.65 | 2.2 / 0.65 | 1.9 / 0.63 | 2.5 / 0.54 | 1.7 / 0.7 | 1.3 / 0.75 | 1.1 / 0.76 | 1.1 / 0.74 | 1.9 / 0.63 | 0.8 / 0.81 |
|  |  |  |  | 84 / 57 | 84 / 56 | 86 / 52 | 80 / 35 | 89 / 67 | 95 / 72 | 95 / 72 | 95 / 70 | 91 / 48 | 98 / 84 |
| 1U7G | 3B9W | 21 / 21 | RBK | 3.5 / 0.50 | 3.4 / 0.51 | 3.2 / 0.53 | 3.4 / 0.50 | 3.0 / 0.61 | 1.5 / 0.63 | 1.5 / 0.65 | 1.4 / 0.64 | 1.4 / 0.63 | 1.1 / 0.74 |
|  |  |  |  | 64 / 35 | 68 / 35 | 70 / 38 | 67 / 34 | 79 / 54 | 86 / 50 | 88 / 54 | 89 / 51 | 86 / 49 | 95 / 72 |
| 3P5N | 4DVE | 21 / 21 | RBK | 3.5 / 0.34 | 3.4 / 0.34 | 4.1 / 0.33 | 3.4 / 0.34 | 4.1 / 0.45 | 2.8 / 0.41 | 2.9 / 0.41 | 2.8 / 0.41 | 2.8 / 0.42 | 1.9 / 0.56 |
|  |  |  |  | 32 / 13 | 30 / 16 | 33 / 15 | 32 / 15 | 57 / 25 | 45 / 20 | 40 / 22 | 42 / 21 | 41 / 18 | 73 / 34 |
| 3P5N | 3RLB | 15 / 13 | RBK | 3.7 / 0.39 | 3.7 / 0.39 | 3.7 / 0.39 | 3.3 / 0.40 | 2.8 / 0.53 | 2.9 / 0.47 | 2.8 / 0.48 | 2.8 / 0.48 | 2.7 / 0.47 | 2.1 (2.6) / 0.63 |
|  |  |  |  | 47 / 20 | 44 / 19 | 46 / 20 | 87 / 45 | 65 / 38 | 59 / 27 | 59 / 27 | 59 / 29 | 58 / 22 | 78 / 49 |
| 3GIA | 4DJK | 15 / 15 | RBK | 4.9 / 0.31 | 4.8 / 0.32 | 4.3 / 0.36 | 4.7 / 0.33 | 4.4 / 0.39 | 3.0 / 0.40 | 3.1 / 0.42 | 3.0 / 0.41 | 3.1 / 0.42 | 3.0 / 0.50 |
|  |  |  |  | 38 / 11 | 40 / 11 | 41 / 16 | 39 / 16 | 45 / 25 | 51 / 16 | 54 / 18 | 52 / 18 | 52 / 23 | 58 / 38 |
| 3ND0 | 3ORG | 20 / 19 | RBK | 2.7 / 0.47 | 2.7 / 0.47 | 4.8 / 0.48 | 2.5 / 0.49 | 2.4 / 0.55 | 1.8 / 0.57 | 1.8 / 0.56 | 4.8 / 0.56 | 1.8 / 0.59 | 1.5 (1.6) / 0.66 |
|  |  |  |  | 65 / 28 | 65 / 28 | 67 / 29 | 69 / 29 | 73 / 40 | 80 / 39 | 80 / 37 | 78 / 39 | 83 / 41 | 90 / 53 |
| 1RH5 | 2ZJS | 21 / 21 | RBK | 4.7 / 0.31 | 4.5 / 0.31 | 6.9 / 0.31 | 4.7 / 0.30 | 5 / 0.39 | 2.5 / 0.52 | 2.4 / 0.53 | 2.5 / 0.52 | 2.5 / 0.50 | 1.9 / 0.6 |
|  |  |  |  | 39 / 15 | 37 / 17 | 38 / 15 | 36 / 15 | 46 / 24 | 69 / 33 | 69 / 32 | 69 / 32 | 66 / 31 | 75 / 45 |
| 3HD6 | 1U7G | 20 / 17 | RBK | 4.6 / 0.44 | 4.5 / 0.45 | 3.0 / 0.52 | 10.6 / 0.29 | 4.0 / 0.49 | 1.9 / 0.59 | 1.8 / 0.59 | 1.6 / 0.59 | 8.9 / 0.39 | 1.5 / 0.65 |
|  |  |  |  | 57 / 29 | 59 / 27 | 67 / 31 | 35 / 14 | 67 / 34 | 82 / 42 | 82 / 41 | 81 / 40 | 50 / 23 | 91 / 50 |
| 3V5U | 4KPP | 18 / 16 | RBK | 5.4 / 0.31 | 5.2 / 0.31 | 5.7 / 0.28 | 5.1 / 0.33 | 4.3 / 0.39 | 3.8 / 0.39 | 3.8 / 0.39 | 4.0 / 0.37 | 3.7 / 0.41 | 3.4 / 0.51 |
|  |  |  |  | 40 / 12 | 40 / 14 | 36 / 11 | 46 /14 | 50 / 26 | 51 / 16 | 51 / 19 | 49 / 17 | 58 / 19 | 70 / 38 |
| 3VVO | 3MKT | 18 / 19 | LR | 2.8 / 0.42 | 2.7 / 0.43 | 3.4 / 0.43 | 2.4 / 0.47 | 2.5 / 0.46 | 2.1 / 0.50 | 2.0 / 0.51 | 2.1 / 0.50 | 1.7 / 0.56 | 2 (2.2) / 0.54 |
|  |  |  |  | 54 / 19 | 54 /18 | 56 / 21 | 62 / 26 | 60 / 23 | 65 / 27 | 69 / 26 | 67 / 25 | 77 / 35 | 75 / 32 |
| 4HZU | 3RLB | 16 / 10 | RBK | 3.1 / 0.44 | 2.9 / 0.45 | 3.2 / 0.43 | 2.6 / 0.44 | 3.7 / 0.49 | 1.8 / 0.55 | 1.9 / 0.56 | 1.8 / 0.56 | 1.8 / 0.56 | 1.9 / 0.61 |
|  |  |  |  | 57 / 22 | 61 / 20 | 55 / 21 | 57 / 20 | 65 / 31 | 75 / 30 | 76 / 29 | 77 / 30 | 76 / 30 | 82 / 43 |
| 4L6R | 4K5Y | 33 / 26 | LR | 3.9 / 0.42 | 4.0 / 0.42 | 4.3 / 0.42 | 3.0 / 0.45 | 3.6 / 0.5 | 2.4 / 0.56 | 2.5 / 0.55 | 2.4 / 0.56 | 1.8 / 0.56 | 1.7 (2.2) / 0.63 |
|  |  |  |  | 53 / 26 | 52 / 26 | 53 / 28 | 57 / 26 | 64 / 38 | 69 / 46 | 69 / 44 | 70 / 45 | 76 / 36 | 83 / 53 |
| 2GFP | 2CFQ | 15 / 12 | RBK | 4.2 / 0.27 | 4.1 / 0.29 | 3.9 / 0.28 | 3.8 / 0.30 | 3.8 / 0.33 | 3.6 / 0.32 | 3.4 / 0.34 | 3.4 / 0.33 | 3.2 / 0.37 | 3.2 / 0.37 |
|  |  |  |  | 26 / 11 | 29 / 12 | 24 / 11 | 33 / 10 | 35 / 13 | 36 / 15 | 37 / 13 | 31 / 16 | 43 / 17 | 38 / 18 |
| 3GD8 | 3KLY | 18 / 17 | LR | 4.9 / 0.33 | 4.6 / 0.34 | 3.2 / 0.44 | 4.5 / 0.34 | 3.5 / 0.46 | 2.1 / 0.52 | 2.0 / 0.52 | 1.9 / 0.54 | 2.0 / 0.51 | 1.7 / 0.64 |
|  |  |  |  | 36 / 13 | 36 / 13 | 54 / 27 | 40 / 12 | 61 / 27 | 64 / 27 | 59 / 24 | 70 / 30 | 63 / 28 | 80 / 48 |
| 3RLB | 3P5N | 15 / 13 | RBK | 4.1 / 0.37 | 10.93 / 0.30 | 4.9 / 0.36 | 3.8 / 0.40 | 4.5 / 0.39 | 2.9 / 0.47 | 7.9 / 0.42 | 2.9 / 0.47 | 2.9 / 0.48 | 2.9 / 0.54 |
|  |  |  |  | 45 / 19 | 35 / 20 | 43 / 19 | 46 / 22 | 41 / 29 | 59 / 27 | 47 / 30 | 53 / 29 | 55 / 30 | 60 / 45 |
