# Supplementary material for: High-Resolution Modeling of Transmembrane Helical Protein Structures from Distant Homologues
Source: PLoS Comput Biol. 2014 May 22;10(5):e1003636. doi: 10.1371/journal.pcbi.1003636 (PMC4031050; doi:10.1371/journal.pcbi.1003636)
Supplement: Table S2 — Improvement of model accuracy in the distorted second TMH of GPCRs. The most accurate among the five lowest energy selected models (see Methods) is reported in the table. a R.m.s. deviation over Cα atoms (in Å) of TMH2 to the crystal structure. b Geometric Distance Test (GDT). This value is the average of four-numbers: the numbers of residues aligned between template or model and crystal structure within 1 Å, 2 Å, 4 Å and 8 Å [48]. c Geometric Distance Test with High-Accuracy (GDT-HA). This value is the average of four-numbers: the numbers of residues aligned between template or model and crystal structure within 0.5 Å, 1 Å, 2 Å and 4 Å [34]. <ΔPHI> and <ΔPSI> represents the average deviation of backbone dihedral angles between template or model and native structure in the de novo rebuilt bend region. (DOCX) [file pcbi.1003636.s003.docx]

**Supplementary Information**

**Chen, K.M., Sun, J., Salvo, J., Baker, D., Barth, P.**

**Supplementary Table 2. Improvement of model accuracy in the distorted second TMH of GPCRs.** The most accurate among the five lowest energy selected models (see Methods) is reported in the table. ^a^ R.m.s. deviation over Cα atoms (in Å) of TMH2 to the crystal structure. ^b^ Geometric Distance Test (GDT). This value is the average of four-numbers: the numbers of residues aligned between template or model and crystal structure within 1 Å, 2 Å, 4 Å and 8 Å [^3^](#_ENREF_3). ^c^ Geometric Distance Test with High-Accuracy (GDT-HA). This value is the average of four-numbers: the numbers of residues aligned between template or model and crystal structure within 0.5 Å, 1 Å, 2 Å and 4 Å [^2^](#_ENREF_2). <ΔPHI> and <ΔPSI> represents the average deviation of backbone dihedral angles between template or model and native structure in the *de novo* rebuild bend region.

| X-ray structure | Receptor template | Receptor template | | | Rosetta | | |
| --- | --- | --- | --- | --- | --- | --- | --- |
|  |  | <ΔPHI> | <ΔPSI> | TM2 (Cα rmsd (Å)^a^ / GDT^b^ / GDT-HA^c^) | <ΔPHI> | <ΔPSI> | TM2 (Cα rmsd (Å)^a^ / GDT^b^ / GDT-HA^c^) |
| 1U19 | 2Z73 | 21.5 | 55.7 | 1.8 / 0.88 / 0.76 | 23.7 | 15.9 | 0.73 / 0.98 / 0.86 |
|  | 3ODU | 21.4 | 15.9 | 0.95 / 0.95 / 0.81 | 16.8 | 13.0 | 0.57 / 0.99 / 0.92 |
| 2RH1 | 2Z73 | 31.6 | 51.9 | 1.8 / 0.87 / 0.74 | 21.9 | 12.3 | 1.1 / 0.93 / 0.82 |
|  | 3ODU | 21.1 | 18.9 | 1.0 / 0.93 / 0.81 | 14.4 | 9.6 | 0.66 / 0.99 / 0.90 |
| 3EML | 1U19 | 32.1 | 51.1 | 1.4 / 0.87 / 0.75 | 5.2 | 4.6 | 0.79 / 0.97 / 0.88 |
|  | 2Z73 | 30.5 | 56.2 | 2.0 / 0.84 / 0.72 | 17.0 | 15.4 | 1.5 / 0.89 / 0.77 |
|  | 3ODU | 25.8 | 16.3 | 1.7 / 0.84 / 0.72 | 16.4 | 7.8 | 1.1 / 0.91 / 0.79 |
| 2Z73 | 1U19 | 25.3 | 57.1 | 1.3 / 0.90 / 0.80 | 26.4 | 46.5 | 1.1 / 0.93 / 0.83 |
|  | 3EML | 37 | 55.4 | 1.8 / 0.87 / 0.75 | 21.9 | 12.4 | 1.0 / 0.92 / 0.80 |
|  | 3ODU | 31.6 | 54.1 | 1.3 / 0.89 / 0.78 | 31.6 | 27.6 | 0.65 / 0.97 / 0.86 |
| 3PBL | 3ODU | 28.3 | 17.3 | 0.93 / 0.95 / 0.81 | 10.7 | 20.2 | 0.59 / 1.00 / 0.88 |
| 3ODU | 2RH1 | 20.8 | 17.3 | 1.1 / 0.92 / 0.80 | 8.8 | 7.2 | 0.76 / 0.98 / 0.87 |
|  | 2Z73 | 25.6 | 54.1 | 1.8 / 0.86 / 0.73 | 16.1 | 12.7 | 1.1 / 0.94 / 0.82 |
|  | 3EML | 73.1 | 43.1 | 2.0 / 0.80 / 0.69 | 15.0 | 10.6 | 1.6 / 0.86 / 0.70 |
| 4DJH | 3UON | 16.8 | 27.0 | 0.83 / 0.95 / 0.87 | 9.2 | 9.9 | 0.72 / 0.96 / 0.88 |
| 3V2Y | 1U19 | 11.5 | 12.9 | 1.4 / 0.90 / 0.77 | 8.2 | 5.1 | 0.88 / 0.93 / 0.87 |
|  | 3ODU | 16.6 | 13.3 | 1.3 / 0.90 / 0.78 | 6.8 | 6.1 | 1.1 / 0.93 / 0.83 |
|  | 3RZE | 19.5 | 8.7 | 1.2 / 0.90 / 0.80 | 5.7 | 5.2 | 0.30 / 1.00 / 0.99 |
| 4EJ4 | 3RZE | 13.8 | 17.8 | 0.90 / 0.94 / 0.86 | 10.4 | 15.1 | 0.68 / 0.96 / 0.86 |
| 3UON | 3ODU | 22.4 | 24.9 | 0.80 / 0.93 / 0.87 | 11.1 | 10.0 | 0.30 / 1.00 / 0.96 |
| 4DAJ | 3ODU | 27.9 | 30.0 | 0.70 / 0.96 / 0.88 | 13.1 | 16.9 | 0.66 / 0.98 / 0.86 |
